# TRIM38 Suppresses Breast Cancer Progression via Modulating SQSTM1 Ubiquitination and Autophagic Flux

**DOI:** 10.1002/advs.202512725

**Published:** 2025-12-05

**Authors:** Shan Jiang, Lijuan Wang, Dianwen Han, Peng Su, Bing Chen, Wenjing Zhao, Tong Chen, Ning Zhang, Xiaolong Wang, Yiran Liang, Yaming Li, Chen Li, Xi Chen, Dan Luo, Qifeng Yang

**Affiliations:** ^1^ Department of Breast Surgery General Surgery Qilu Hospital of Shandong University Jinan Shandong 250012 P. R. China; ^2^ Biological Resource Center Qilu Hospital of Shandong University Jinan Shandong 250012 P. R. China; ^3^ Department of Pathology Qilu Hospital of Shandong University Jinan Shandong 250012 P. R. China; ^4^ Research Institute of Breast Cancer Shandong University Jinan Shandong 250012 P. R. China

**Keywords:** autophagic flex, breast cancer, SQSTM1, TRIM38, ubiquitination

## Abstract

TRIM38, an E3 ubiquitin‐protein ligase, has previously been implicated in innate immune and inflammatory responses, yet its role in breast cancer regulation remains unclear. This study elucidates the suppressive function of TRIM38 in breast cancer progression. The results indicate a decreased expression of TRIM38 in breast cancer tissues compared to adjacent non‐cancerous counterparts, and its reduced expression correlates with unfavorable clinical outcomes in breast cancer patients. Both in vitro and in vivo experiments demonstrate that TRIM38 inhibits breast cancer proliferation, migration, and invasion. Furthermore, an inverse regulatory relationship between TRIM38 protein level and autophagic flux is observed. Mechanistically, SQSTM1/p62 is identified as a novel substrate of TRIM38, which promotes non‐degradative K63‐linked ubiquitination at SQSTM1 K420 residue. This kind of ubiquitination disrupts the interaction between SQSTM1 and LC3, thereby impeding autophagic flux. Collectively, the findings underscore TRIM38 as a crucial regulator of autophagy and present novel, promising therapeutic targets for breast cancer.

## Graphical abstract

TRIM38, an E3 ubiquitin ligase, suppresses breast cancer progression by inhibiting proliferation, migration, and invasion. Downregulated in breast tumor, its loss correlates with poor prognosis. Mechanistically, TRIM38 mediates K63‐linked ubiquitination of SQSTM1/p62 at K420, disrupting SQSTM1‐LC3 interaction and blocking autophagic flux. These findings highlight TRIM38 as a key autophagy regulator and a potential therapeutic target.

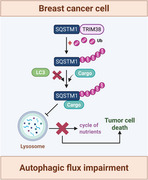

## 1 Introduction

Breast cancer represents the second leading cause of cancer incidence among women globally.^[^ ^1^ ^]^ However, the exact mechanisms leading to its occurrence and development remain poorly understood. It has been reported that several important cellular signaling pathways, such as ubiquitylation and autophagy, among others, participate in the processes of breast cancer proliferation and metastasis.^[^ ^2^, ^3^ ^]^ The in‐depth studies of key molecules in these pathways would be helpful to clarify the mechanism underlying breast cancer progression, thereby facilitating potential targets and ultimately developing effective targeted therapies for breast cancer patients.

TRIM38, a member of tripartite motif (TRIM) family, functions as an E3 ubiquitin‐protein ligase predominantly localized in cytoplasm.^[^ ^4^ ^]^ It shares the conserved domains of RING, B‐box, coiled‐coil (CC), and PRY/SPRY with other TRIM family proteins and facilitates K48‐ and K63‐linked ubiquitination through its RING domain.^[^ ^5^ ^]^ Initially recognized for its role in innate immunity, TRIM38 exhibits context‐dependent regulatory functions, suppressing antiviral responses by competing with TRIM25 for RIG‐I binding to inhibit type I interferon production, while enhancing RIG‐I/MDA5 activation to exhibit antiviral activity.^[^ ^6^, ^7^ ^]^ Beyond antiviral defense, TRIM38 negatively regulates inflammatory signaling by targeting distinct adaptor proteins: it degrades NAP1 and TRIF to suppress TLR3/TLR4‐driven NF‐κB activation, induces TAB2/3 degradation to inhibit TNFα/IL‐1β‐induced pathways, and eliminates TRAF6 to attenuate TLR7/9‐mediated responses. Furthermore, it sustains immune homeostasis through SUMOylation of cGAS, thereby calibrating interferon production ^[^ ^8^, ^9^, ^10^, ^11^, ^12^ ^]^ In cellular physiology, TRIM38 regulates cell differentiation and survival, driving M2 macrophage polarization via K63‐linked ubiquitination of HSPA5, and promotes osteoblast function while protecting chondrocytes from IL‐1β‐induced apoptosis via NF‐κB inhibition.^[^ ^13^, ^14^, ^15^ ^]^ Emerging evidence highlights its tumor‐suppressive roles, including downregulates GLUT1 in bladder cancer, inhibits AMPK/NF‐κB/NLRP3 pathway in non‐small cell lung cancer, and suppresses CCT6A ubiquitination‐mediated MYC pathway in colorectal cancer.^[^ ^16^, ^17^, ^18^ ^]^ However, the precise function of TRIM38 in breast cancer progression remains elusive.

Autophagy participates in the regulation of diverse tumor behaviors, serves as a double‐edged sword in preventing early‐stage tumor development and maintaining the metabolic adaptation of established or metastatic tumors.^[^ ^19^ ^]^ It has always been considered as a target for drug blockade in clinical practice.^[^ ^20^ ^]^ Sequestosome‐1 (SQSTM1)/p62 is a common cargo receptor, which is of vital importance in multiple stages of autophagy processes.^[^ ^21^ ^]^ After binding ubiquitinated proteins via its UBA domain, SQSTM1 attaches to LC3 on the autophagosome membrane through its LIR domain. Subsequently, this complex is continuously degraded by non‐selective autophagy, thereby maintaining the SQSTM1 content in a dynamic equilibrium.^[^ ^22^, ^23^ ^]^ In addition to its involvement in autophagy pathway, SQSTM1 is also a multifunctional signaling regulator implicated in apoptotic cell death and oxidative stress response.^[^ ^24^, ^25^ ^]^ In the progression of breast cancer, SQSTM1 appears to play a complex role. By participating in the autophagic degradation of certain oncogenic proteins, SQSTM1 can suppress breast cancer motility and remodel the tumor microenvironment.^[^ ^26^, ^27^ ^]^ Conversely, the accumulation of SQSTM1 may enhance tumor stemness and decrease tumor chemosensitivity by increasing the stability of carcinogenic mRNAs and the transcription of oncogenic genes.^[^ ^28^, ^29^ ^]^ Hence, the role of SQSTM1 in cellular biological processes and the regulatory mechanisms governing its content and function in breast cancer are worth to be studied.

In this study, we observed that TRIM38 expression level of tumor specimens is intensively correlated with the prognosis in breast cancer patients. Moreover, both in vivo and in vitro experiments demonstrated that TRIM38 could suppress the growth, migration, and invasive capacity of breast cancer cells, indicating its suppressive role in cancer progression. Mechanistically, our study revealed TRIM38 as a previously unrecognized E3 ligase for SQSTM1/p62, catalyzing K63‐linked polyubiquitination specifically at its K420 residue. This post‐translational modification sterically hindered SQSTM1‐LC3 complex formation. Consequently, TRIM38 impeded autophagic flux and the circulation of cytoplasmic substances, leading to the impairment of breast cancer progression. Overall, our findings suggest a potential therapeutic strategy targeting the TRIM38/SQSTM1/autophagic flux axis for breast cancer treatment.

## 2 Results

### 2.1 Low Expression of TRIM38 Correlates with Poor Prognosis in Breast Cancer Patients

**Figure 1:**
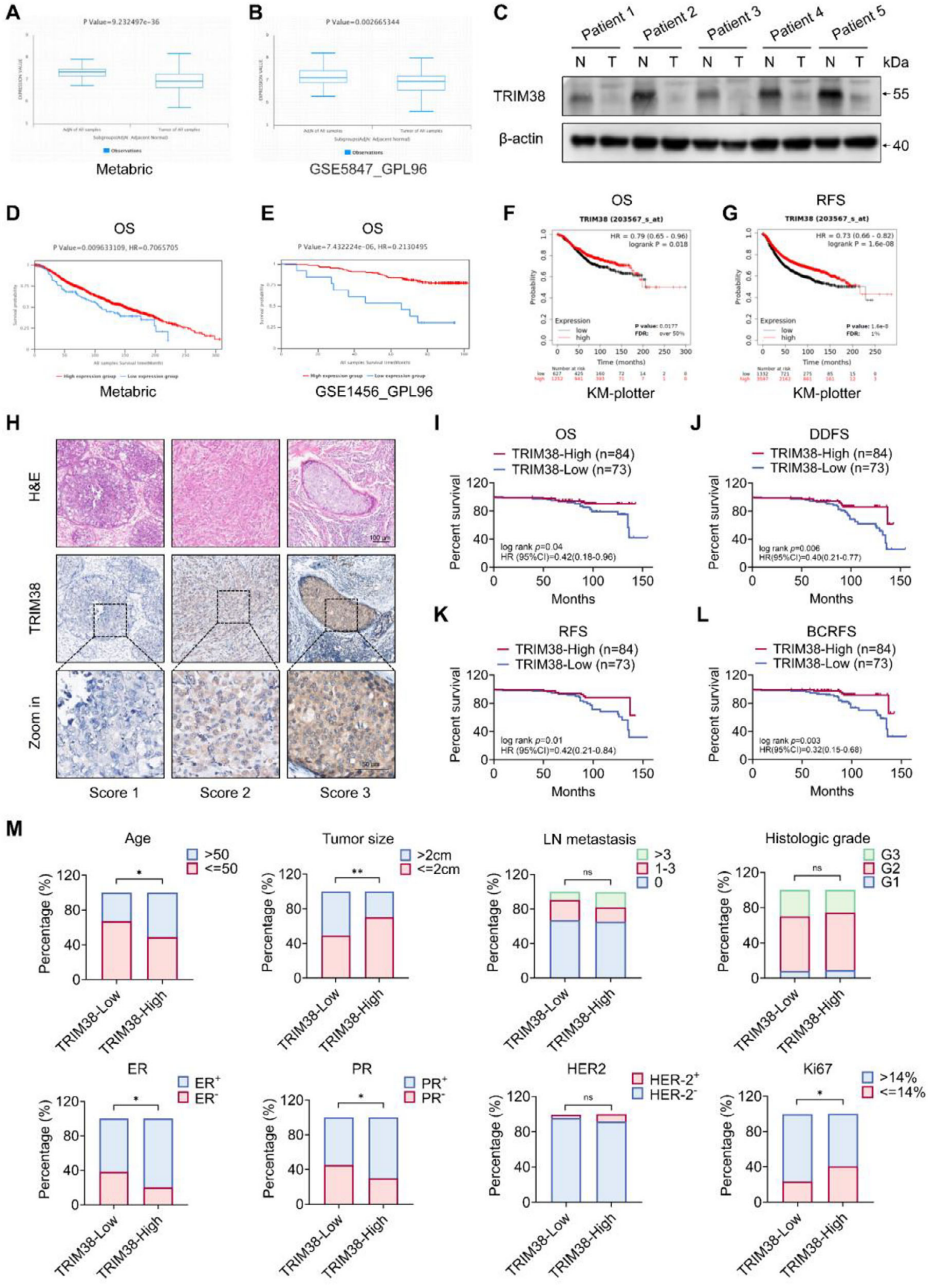
Low expression of TRIM38 correlates with poor prognosis in breast cancer patients. A,B) TRIM38 expression level obtained from BCIP online database. Sample number(normal/tumor): A = 2124(144/1980), B = 95(47/48). C) Western blotting of cancer tissue protein obtained from breast cancer patients (N = adjacent non‐cancerous tissues, T = tumor tissue). D,E) Kaplan–Meier curves got from BCIP about TRIM38 expression level and prognosis. Sample number(normal/tumor): D = 2124(144/1980), E = 157(0/157). F,G) Kaplan–Meier curves got from Kaplan–Meier Plotter database about TRIM38 expression level and prognosis. H) H&E staining for tumor tissues and typical IHC images of the TRIM38 protein expression in carcinoma regions of breast cancer specimen. I–L) Patient OS (I), DDFS (J), RFS(K), and BCRFS(L) analysis between TRIM38‐high/low groups based on the IHC score. Differences in patient survival outcomes were compared using Kaplan–Meier curves and log‐rank tests. M) Clinicopathologic features as determined in the TRIM38‐high/low groups based on the IHC score. *p*‐values were determined via two‐tailed chi‐squared tests. *p < 0.05; **p < 0.01; ***p < 0.001; ns, no significance.

To evaluate the role TRIM38 plays in breast cancer and assess its potential clinical value, we performed comprehensive analyses using both publicly available datasets and our own collected clinical samples and patient data. Initially, we utilized the BCIP online database (http://www.omicsnet.org/bcancer/database) to investigate the changes of TRIM38 expression in breast cancer. Data from both Metabric database and GSE5847_GPL96 consistently showed downregulated TRIM38 expression in breast cancer tissues compared to adjacent non‐cancerous tissues (up to 1 cm from tumor margins), which was historically designated as clinically normal counterparts ^[^ ^30^ ^]^ (**Figure** **1**A,B). Tissue protein extracted from clinical specimens exhibited a similar trend, as depicted in Figure 1C. Subsequently, we utilized both BCIP and Kaplan–Meier Plotter database (https://kmplot.com/analysis) to find out the association between TRIM38 expression level and patient prognosis.^[^ ^31^, ^32^ ^]^ The public data revealed that a low level of TRIM38 expression in breast cancer tissues was associated with a poor prognosis for patients (Figure 1D–G).

Beyond online data, we further validated TRIM38 abundance through immunohistochemistry (IHC) in 157 formalin‐fixed, paraffin‐embedded (FFPE) breast cancer specimens obtained from Qilu Hospital with tumor verification via H&E staining. The demographics and clinicopathologic characteristics information of 157 patients is detailed in Table S2 (Supporting Information). For stratification, TRIM38 IHC scores were generated by combining staining intensity with the percentage of positively stained carcinoma regions (including both in situ and invasive components). Patients were subsequently divided into TRIM38‐high and ‐low subgroups based on the IHC score. Representative H&E staining and IHC staining images are shown in Figure 1H. Survival analysis demonstrated that decreased TRIM38 protein levels correlated with shortened overall survival (OS), disease‐free survival (DDFS), recurrence‐free survival (RFS), and 5‐year breast cancer recurrence‐free survival (BCRFS) (Figure 1I–L). Given the biochemical heterogeneity of breast cancer, we then stratified patients by subtypes (HR^+^/HER2^+^, HR^+^/HER2^−^, HR^−^/HER2^−^; excluding HR^−^/HER2^+^) and separately evaluated TRIM38's impact on OS and DDFS (Figure S1A–F, Supporting Information). However, our results demonstrated that elevated TRIM38 expression was significantly associated with prolonged DDFS exclusively in HR^+^/HER2^−^ patients, whereas no statistically meaningful differences were detected in other molecular subtypes. We speculate that this difference might be due to the limitations in sample selection: the proportion of HR^+^/HER2^−^ cases in our study sample was excessively high, which may reflect the selection bias. Additionally, the sample sizes of the HR^+^/HER2^+^ and HR^−^/HER2^−^ subgroups were insufficient, thereby reducing the statistical power when assessing the correlation. Moreover, the absence of HER2^−^enriched (HER2^−^/HR^+^) patients in our sample might have introduced selection bias. Future studies will expand enrollment across all subtypes and employ enhanced statistical modeling to validate TRIM38's subtype‐specific survival correlations. Clinicopathological characteristics associated with TRIM38 expression were summarized in Supplementary Table 3. Notably, lower TRIM38 expression was associated with younger age, larger tumor size, lower expression of estrogen and progesterone receptors (ER and PR, respectively), and higher Ki67 expression (Figure 1M). However, TRIM38 expression level showed no significant in lymph node metastasis number, histological grade and HER2 expression (Figure 1M). As presented in Tables S4 and S5 (Supporting Information), univariate and multivariate Cox proportional hazards regression analyses revealed that TRIM38 expression level emerged as an independent predictor of OS and DDFS in breast cancer patients.

### 2.2 TRIM38 Exhibits Tumor‐Suppressive Effects

**Figure 2:**
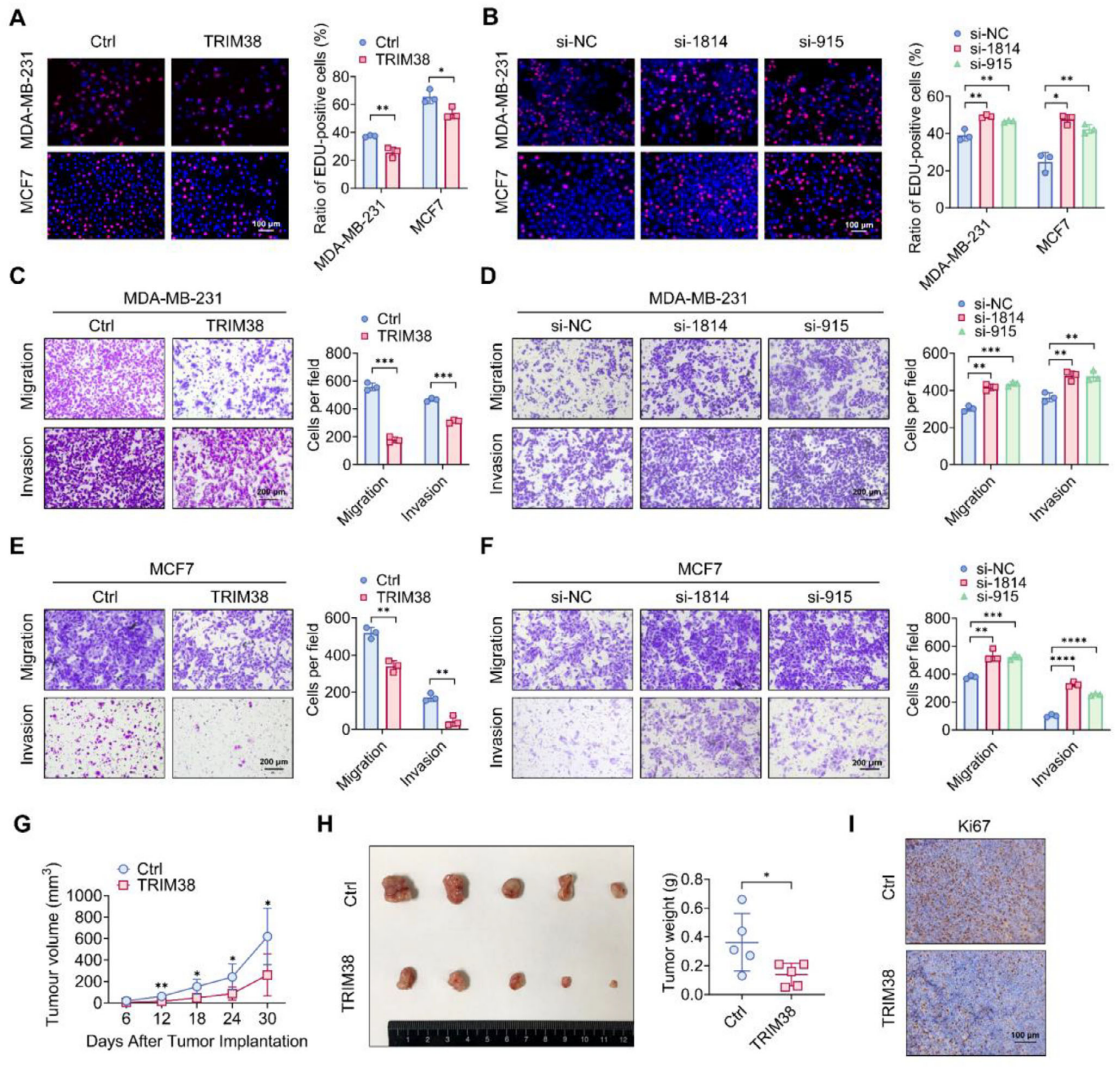
TRIM38 exhibits tumor‐suppressive effects. A) EdU assays were conducted in MDA‐MB‐231 and MCF7 with transfection of TRIM38 plasmid. B) EdU assays were conducted in MDA‐MB‐231 and MCF7 after being transfected with si‐TRIM38. C,D) Transwell assays of MDA‐MB‐231 transfected with TRIM38 plasmid (C) or si‐TRIM38 (D). E,F) Transwell assays of MCF7 transfected with TRIM38 plasmid (E) or si‐TRIM38 (F). G,H) MDA‐MB‐231 with or without endogenous TRIM38 overexpression was implanted on BALB/c‐nude (n = 5). Tumor growth curve (G), tumor images and tumor weight (H) of the aforementioned group were as shown. I) Immunohistochemical staining for Ki67 among the indicated groups. Significance was determined by unpaired two‐tailed Student's *t*‐test: *p < 0.05; **p < 0.01; ***p < 0.001, ****p < 0.0001. All experiments were repeated at least thrice and the results of representative experiments are shown.

To elucidate the functional significance of TRIM38 in breast cancer progression, we employed an overexpression plasmid and TRIM38‐specific siRNAs, named si‐1814 and si‐915, for efficient overexpression and knockdown of TRIM38 in vitro, respectively. EdU assays (**Figure** **2**A), MTT assays (Figure S2A,B, Supporting Information), and cell plate colony formation assays (Figure S2E,F, Supporting Information) demonstrated that overexpression of TRIM38 significantly attenuated the growth ability of breast cancer cell lines. Conversely, breast cancer cells with TRIM38 interference exhibited a higher proliferation rate (Figure 2B; Figure S2C–F, Supporting Information). The chemotactic‐driven and collective migratory and invasive capacities of breast cancer cells in vitro were quantitatively assessed using transwell and wound healing assays. Our data indicated that high level of TRIM38 expression was associated with reduced migratory and invasive abilities in breast cancer cells (Figure 2C,E; Figure S2G,I, Supporting Information) and TRIM38 interference enhanced these aggressive phenotypes (Figure 2D,F; Figure S2H,J, Supporting Information), suggesting a critical role in suppressing metastatic progression potentially. To further investigate the effect of TRIM38 on tumor growth in vivo, we first validated the specificity of qPCR primers (Figure S3A–C, Supporting Information) and then established an MDA‐MB‐231 cell line stably overexpressing TRIM38, with overexpression efficiency confirmed by RT‐PCR and western blotting (Figure S3D,E, Supporting Information). These cells were then utilized for subsequent xenograft assays. In xenograft tumor experiments, MDA‐MB‐231 cells with high TRIM38 expression showed a slower growth rate (Figure 2G), resulting in a smaller tumor weight compared to the control group (Figure 2H). Ki67 is a nuclear biomarker commonly used for evaluating cellular proliferation in breast cancer.^[^ ^33^ ^]^ Our results showed that immunohistochemical staining of Ki67 was decreased in the TRIM38‐overexpression group (Figure 2I), suggesting a tumor‐suppressive effect produced by high level of TRIM38 in vivo. Collectively, our results indicate that TRIM38 suppresses breast cancer malignancy behaviors in both cellular and animal models.

### 2.3 TRIM38 Interacts with SQSTM1

**Figure 3:**
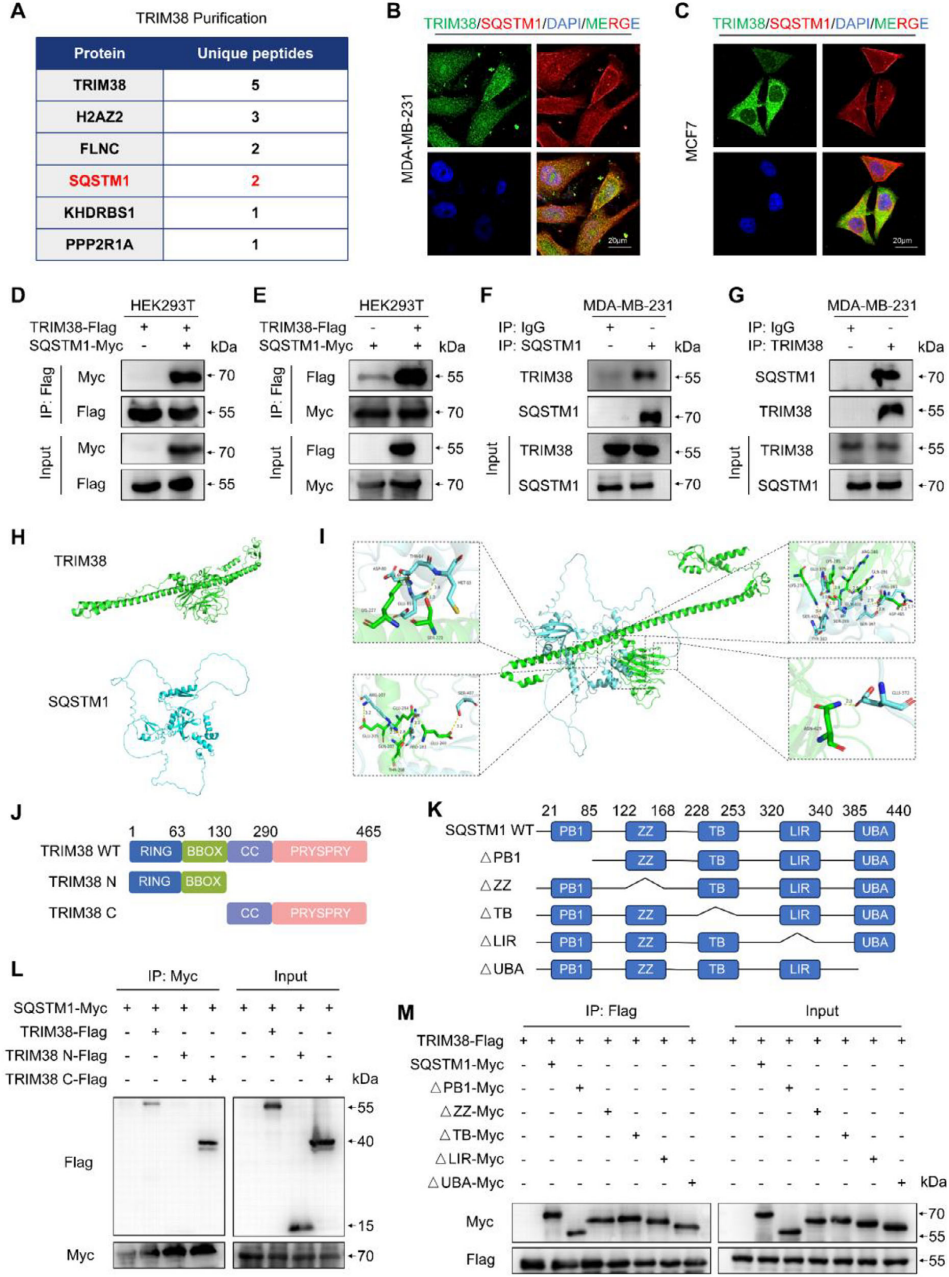
TRIM38 interacts with SQSTM1. A) Top five high‐confidence interactors of TRIM38 identified through LC‐MS/MS analysis, with rabbit IgG immunoprecipitation‐based background subtraction. B,C) Confocal microscopy assay to assess the endogenous co‐localization of TRIM38 and SQSTM1 in MDA‐MB‐231 (B) or MCF7 cells (C). D,E) Exogenous Co‐IP assays of the interaction between TRIM38 and SQSTM1 after transiently co‐transfection of Flag‐tagged TRIM38 and Myc‐tagged SQSTM1 in HEK293T cells. F,G) Endogenous Co‐IP analysis of the interaction between the TRIM38 and SQSTM1 in MDA‐MB‐231 cells, with Rabbit IgG serving as a control. H) 3D molecular structure of TRIM38 and SQSTM1 predicted by Alphafold3. I) Molecular docking model of the combination between TRIM38 and SQSTM1 predicted by GRAMM docking. J) Schema graphs of TRIM38 truncated mutants. K) Schematic diagrams of SQSTM1 truncated mutants. L) HEK293T cells were transfected with Myc‐SQSTM1 and Flag‐TRIM38 truncated mutants. Extracts were subjected to IP with anti‐Myc, followed by Western blotting with anti‐Flag and anti‐Myc. M) HEK293T cells were transfected with Flag‐TRIM38 and a panel of Myc‐tagged SQSTM1‐truncated mutant plasmids. IP assays were conducted to evaluate interactions between TRIM38 and the SQSTM1‐truncated mutants. All experiments were repeated at least three times and the results of representative experiments are shown.

To identify potential downstream targets of TRIM38 in breast cancer, we immunoprecipitated TRIM38 in MDA‐MB‐231 cells, and the precipitated complexes were subjected to liquid chromatography tandem mass spectrometry (LC‐MS/MS) analysis after verification of immunoprecipitation efficiency (Figure S4A, Supporting Information). Among the proteins identified by LC‐MS analysis with a relatively high number of unique peptides, SQSTM1 emerged as a potential binding partner of TRIM38 with rabbit IgG immunoprecipitation‐based background subtraction (**Figure** **3**A; Figure S4B, Supporting Information). Confocal analysis subsequently demonstrated colocalization between endogenous TRIM38 and SQSTM1 in breast cancer cell lines (Figure 3B,C). To further validate the interaction between TRIM38 and SQSTM1, exogenous IP assays were conducted. The results confirmed that SQSTM1 co‐precipitated with TRIM38 in HEK293T cells (Figure 3D), and reciprocally, TRIM38 also co‐precipitated with SQSTM1 (Figure 3E). In MDA‐MB‐231 cells, endogenous TRIM38 was specifically co‐immunoprecipitated with anti‐SQSTM1 antibody but not a control IgG (Figure 3F). Correspondingly, endogenous SQSTM1 was also co‐immunoprecipitated with anti‐TRIM38 antibody, but not with a control IgG (Figure 3G). To gain insights into the specific domains required for this interaction, we employed AlphaFold3 to predict the spatial structures of the two proteins (Figure 3H). The molecular docking model produced by GRAMM docking software revealed potential binding sites between TRIM38 and SQSTM1 (Figure 3I). Based on this model, we constructed two Flag‐tagged TRIM38 truncation mutants (Figure 3J) and a panel of Myc‐tagged SQSTM1 truncation mutants (Figure 3K). Co‐IP experiments indicated that the CC and PRY/SPRY domain of TRIM38 were essential for its interaction with SQSTM1 (Figure 3L). Interestingly, the deletion of any single domain in SQSTM1 did not disrupt its binding with TRIM38, which was consistent with the docking model, suggesting a multi‐sited interaction between SQSTM1 and TRIM38 tightly (Figure 3M).

### 2.4 TRIM38 Promotes SQSTM1 K63 Polyubiquitination at K420 Residue

**Figure 4:**
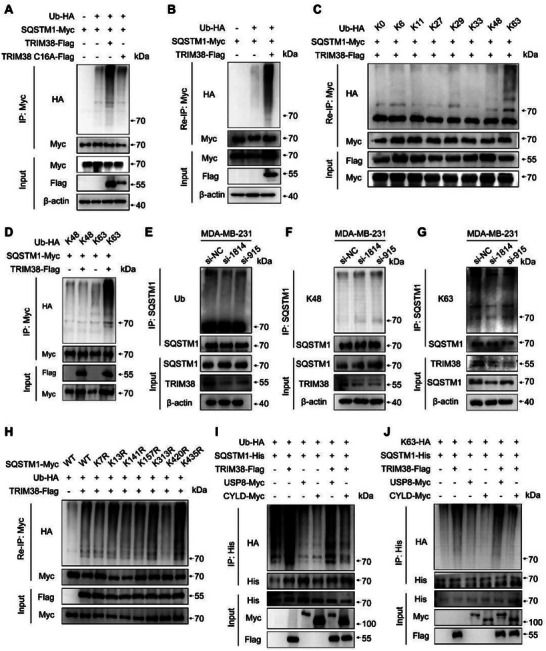
TRIM38 promotes SQSTM1 K63 polyubiquitination at K420 residue. A) Lysates from HEK293T cells were transiently co‐transfected with Myc‐SQSTM1, HA‐Ub, Flag‐TRIM38, and Flag‐TRIM38 C16A, followed by immunoprecipitation using anti‐Myc and immunoblotting using anti‐HA. B) Re‐IP assay was conducted in lysates from HEK293T cells after co‐transfection with Myc‐SQSTM1, HA‐Ub and Flag‐TRIM38, and then analyzed by Western blotting. C) After co‐transfected with HA‐Ub (WT and its mutants), Flag‐TRIM38, and Myc‐SQSTM1, lysates from HEK293T cells were immunoprecipitated twice with anti‐Myc, following by immunoblotting using anti‐HA. D) Immunoprecipitation analysis of lysates from HEK293T cells after transient co‐transfection of Myc‐SQSTM1, Flag‐TRIM38, and HA‐K48 or HA‐K63. E–G) After endogenous TRIM38 interference, lysates from MDA‐MB‐231 were co‐immunoprecipitated by anti‐SQSTM1, and level of total ubiquitin (E), K48‐linked ubiquitin (F) and K63‐linked ubiquitin (G) in immunocoprecipitate were detected by Western blotting. H) Re‐IP analysis was performed in lysates from HEK293T cells co‐transfected with HA‐Ub, Flag‐TRIM38 and a serious of Myc‐SQSTM1 point mutant plasmids. I,J) HEK293T cells were co‐transfected with His‐SQSTM1, Flag‐TRIM38, Myc‐USP8/Myc‐CYLD and HA‐Ub (I) /HA‐K63 (J), following immunoprecipitation using anti‐His and western blotting. All experiments were repeated at least thrice and the results of representative experiments are shown.

Considering the function as an E3 ubiquitin ligase of TRIM38, we conducted a series of assays to investigate its effects on SQSTM1 ubiquitination. Ubiquitin mutant vectors K48 and K63 were constructed with all lysine (K) residues replaced by arginine (R), except for the 48th or 63rd residues, respectively. Additionally, ubiquitin mutant vectors K6R, K11R, K27R, K29R, K33R, K48R, and K63R harbored an only arginine mutation at the respective lysine residues. These vectors were employed to elucidate the form of polyubiquitination mediated by TRIM38. Our findings revealed that TRIM38 significantly enhanced both total ubiquitination and K63‐linked ubiquitination of SQSTM1 (**Figure** **4**A,D,E,G; Figure S5A, Supporting Information). Conversely, TRIM38 had no impact on K48‐linked ubiquitination, either endogenously or exogenously (Figure 4D,F; Figure S5A, Supporting Information). Notably, the TRIM38 C16A mutant, which is devoid of ubiquitin ligase activity due to a cysteine‐to‐alanine substitution, exhibited no effects on SQSTM1 ubiquitination (Figure 4A). To minimize interference from nonspecifically conjugated protein, a two‐step IP (Re‐IP) assay was conducted as previously reported.^[^ ^34^, ^35^, ^36^ ^]^ HEK293T cells were co‐transfected with Myc‐tagged SQSTM1, HA tagged‐ubiquitin and Flag tagged‐TRIM38. Cell lysates were then subjected to IP with anti‐Myc antibody, and then the immunoprecipitates were denatured and subjected to sequential re‐IP with anti‐Myc antibody again. Notably, total ubiquitination and K63‐linked ubiquitination of SQSTM1 were markedly elevated under the presence of TRIM38 (Figure 4B,C).

Furthermore, we investigated the ubiquitylation site of SQSTM1 mediated by TRIM38. Potential ubiquitylation sites on SQSTM1 were predicted using GPS‐Uber (https://gpsuber.biocuckoo.cn/online.php) (Figure S5B, Supporting Information). Based on the predictions, we generated seven Myc‐tagged SQSTM1 plasmids with a lysine‐to‐arginine mutation at K7, K13, K141, K157, K313, K420, and K435, respectively, rendering these residues unable to undergo ubiquitination at the respective sites. Subsequent Re‐IP assays demonstrated that the K420R mutation disrupted ubiquitination of SQSTM1 mediated by TRIM38 (Figure 4H). Previous studies have reported that USP8 could directly remove K11‐linked ubiquitination of SQSTM1 at K420,^[^ ^37^ ^]^ while K63‐linked‐specific deubiquitinase CYLD could interact with SQSTM1, leading to its K63‐linked deubiquitylation.^[^ ^38^, ^39^ ^]^ To further confirm that TRIM38 adds K63‐linked polyubiquitin chains at SQSTM1 K420 residue, we conducted co‐transfection experiments utilizing USP8 and CYLD. Figure 4I shows that USP8 could partially remove wild‐type ubiquitin added by TRIM38, and CYLD could completely counteract the ubiquitination of SQSTM1 mediated by TRIM38. However, when wild‐type ubiquitin was replaced with K63, USP8 was unable to remove polyubiquitin added by TRIM38, whereas CYLD could still offset the ubiquitination of SQSTM1 mediated by TRIM38 (Figure 4J). These results conclusively demonstrated that TRIM38 added K63‐linked polyubiquitin at SQSTM1 K420 residue.

### 2.5 TRIM38 Inhibits the Degradation of SQSTM1 via Lysosome Pathway

**Figure 5:**
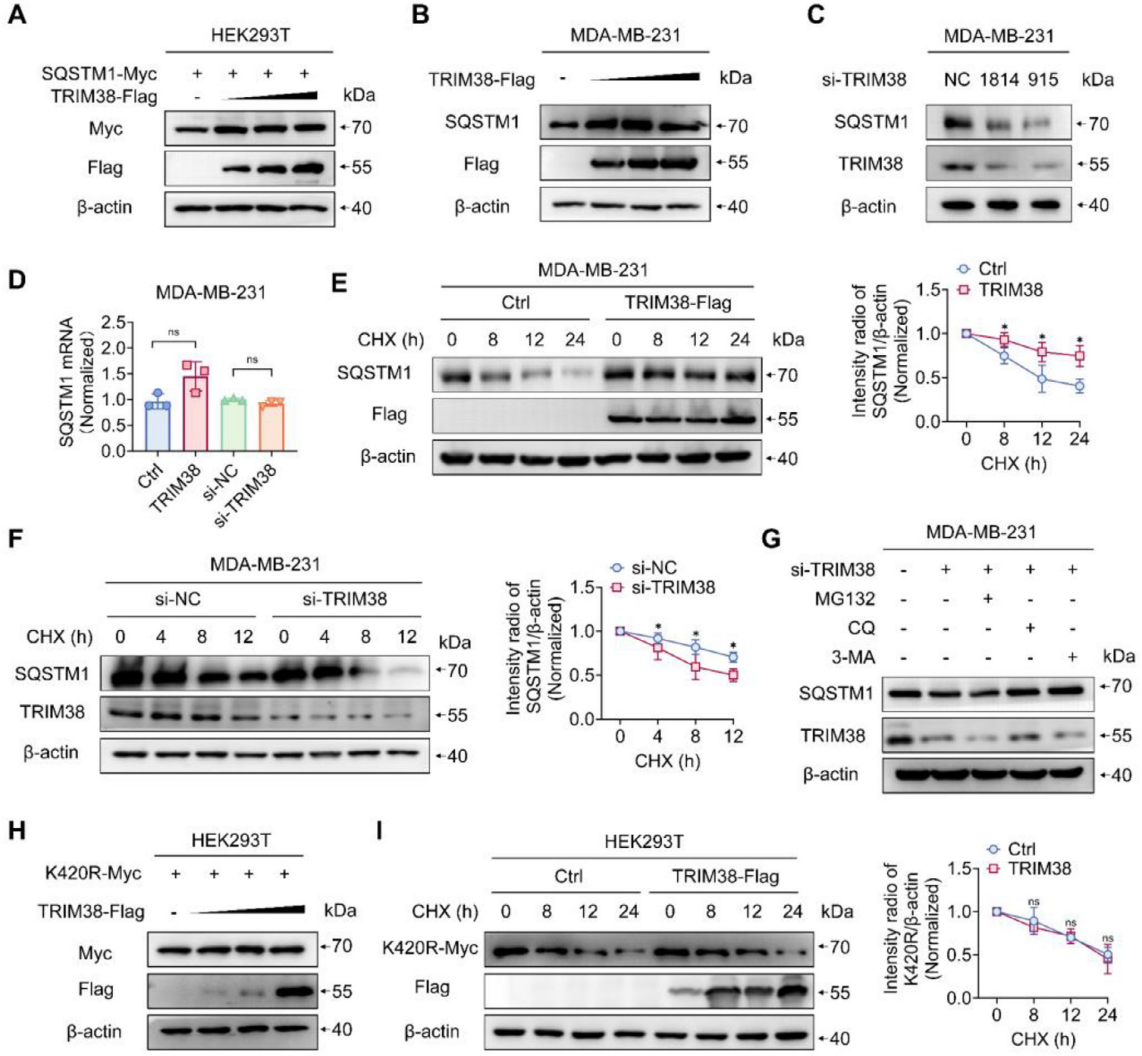
TRIM38 impedes SQSTM1 degradation by lysosome pathway. A) Western blotting of extraction from HEK293T with transfection of Myc‐SQSTM1 and dose‐increased Flag‐TRIM38. B) Immunoblotting of lysates from MDA‐MB‐231 cells transfected with a gradually increasing dose of Flag‐TRIM38. C) Western blotting of lysates from MDA‐MB‐231 cells transfected with si‐NC or two TRIM38 siRNAs for 48 hours. D) RT‐qPCR examination of SQSTM1 mRNA expression following transfection with TRIM38‐overexpressing plasmids or TRIM38 siRNA. E,F) Western blotting analysis of SQSTM1 expression in MDA‐MB‐231 cells transfected with Flag‐TRIM38 (E) or TRIM38 siRNA (F) followed by treatment with cycloheximide (CHX, 10 µm) for the indicated periods. Band intensities, measured using “ImageJ” software, represent protein expression levels. G) Immunoblotting of lysates from MDA‐MB‐231 cells transfected with si‐TRIM38 and treated with MG132 (10 µm), chloroquine (CQ, 100 µm), or 3‐MA (5 mm) for 6 h. H) Western blotting of lysates from HEK293T cells transfected with Myc‐SQSTM1 K420R and an increased dose of Flag‐TRIM38. I) Immunoblot analysis of lysates from HEK293T cells transfected with Flag‐TRIM38 and Myc‐SQSTM1 K420R and treated with CHX for the indicated durations. Protein expression intensities were normalized to β‐actin. Significance was determined by unpaired two‐tailed Student's *t*‐test: *p < 0.05; ns, no significance. All experiments were repeated at least thrice and the results of representative experiments are shown.

Since K63‐linked ubiquitination may lead to alterations in substrate content,^[^ ^40^ ^]^ we examined whether TRIM38 could regulate SQSTM1 expression. Strikingly, the protein levels of SQSTM1 and TRIM38 showed a positive correlation, displaying an “on‐off” switch‐like pattern rather than a dose‐dependent relationship (**Figure** **5**A,B). Endogenous interference of TRIM38 also decreased the content of SQSTM1 in tumor cells (Figure 5C). Nevertheless, neither overexpression nor interference of TRIM38 affected SQSTM1 expression at mRNA level (Figure 5D). Therefore, we hypothesized that TRIM38 could augment the intracellular content of SQSTM1 by impeding its degradation. CHX assays demonstrated that TRIM38 could decelerate the degradation rate of SQSTM1 and enhance its protein stability (Figure 5E,F). Subsequently, we employed various inhibitors to further explore the pathway through which TRIM38 affects SQSTM1 stability. The proteasome inhibitor MG132 is a commonly used 26S proteasome that blocks the proteolytic activity of this complex, thereby preventing the degradation of ubiquitinated proteins,^[^ ^41^, ^42^, ^43^ ^]^ had no effect on TRIM38‐induced SQSTM1 stability (Figure 5G). Meanwhile, chloroquine (CQ), which inhibits lysosome activity via blocking autophagosome formation and impairing lysosomal acidification,^[^ ^44^, ^45^ ^]^ and 3‐methyladenine (3‐MA), which inhibits PI3K activity and therefore inhibits autophagy,^[^ ^46^ ^]^ could reverse SQSTM1 degradation induced by TRIM38 interference (Figure 5G). However, the protein level of SQSTM1 K420R was unaffected by TRIM38 overexpression (Figure 5H). And the stability of SQSTM1 K420R could not be affected by TRIM38 either (Figure 5I). Collectively, these results indicated that TRIM38‐mediated ubiquitination of SQSTM1 prevented its degradation via the lysosome pathway.

### 2.6 TRIM38 Negatively Regulates Autophagy Flux

**Figure 6:**
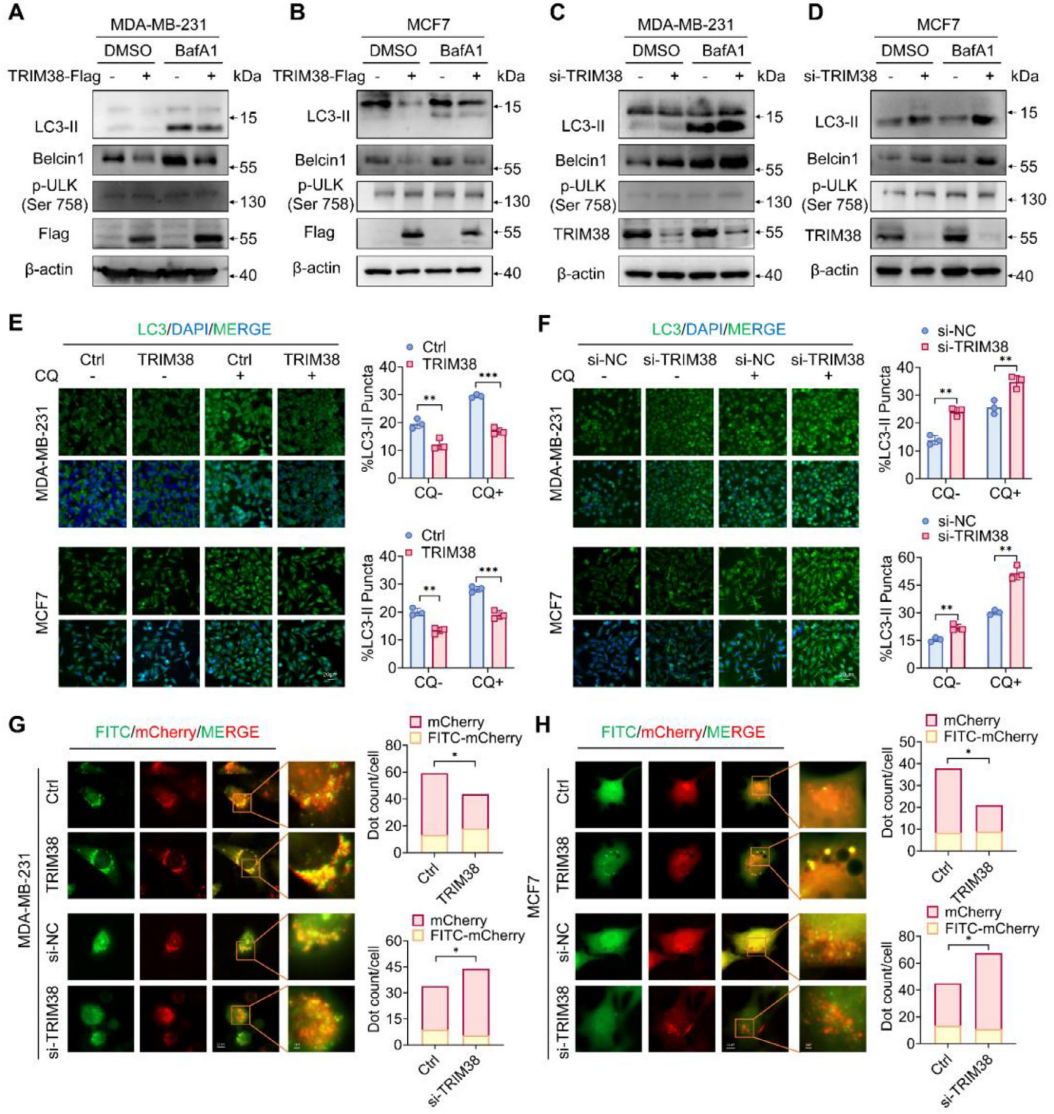
TRIM38 negatively regulates autophagy flux. A,B) Immunoblotting of lysates from MDA‐MB‐231 (A) and MCF7 (B) cells transfected with Flag‐TRIM38 or empty vector control after the treatment with bafilomycin A1 (BafA1, 200 nm) or vehicle control for 6–8 h. C,D) MDA‐MB‐231 (C) and MCF7 (D) cells underwent the aforementioned treatment after transfection of si‐TRIM38 or si‐NC. E) Immunofluorescence of LC3 in MDA‐MB‐231 and MCF7 cells transfected with TRIM38‐overexpressing plasmid or empty vectors after being treated with or without CQ for 8 h. The proportion of cells containing LC3 puncta was quantified using “ImageJ”. F) Immunofluorescence of LC3 in MDA‐MB‐231 and MCF7 cells underwent the same treatment after transfection of si‐TRIM38 or si‐NC. G,H) Fluorescence microscopy analysis of MDA‐MB‐231 (G) and MCF7 (H) cells infected with mCherry‐GFP‐LC3B adenovirus for 48–96 hours following 24‐hour transfection with either Flag‐TRIM38 or si‐TRIM38, using a Zeiss Axio Observer 7 microscope. The number of red or yellow dots was counted with “ImageJ”. Significance was determined by unpaired two‐tailed Student's *t*‐test: *p < 0.05; **p < 0.01; ***p < 0.001. All experiments were repeated at least three times and the results of representative experiments are shown.

Previous studies have reported that ubiquitination of SQSTM1 K420 residue was associated with the regulation of autophagy.^[^ ^37^, ^47^, ^48^ ^]^ Therefore, we conducted a series of experiments to assess the potential influence of TRIM38 on autophagic processes. Notably, TRIM38 overexpression significantly decreased LC3‐II and Beclin1 in breast cancer cells under both the presence or absence of the lysosome inhibitor bafilomycin A1 (BafA1), which blocks the fusion of autophagosomes with lysosomes by inhibiting V‐ATPase activity, therefore impedes late autophagy (**Figure** **6**A,C). And TRIM38 overexpression showed little significant effect on ULK1 phosphorylation levels, suggesting its limited regulatory role in autophagy initiation (Figure 6A,C). Conversely, TRIM38 knockdown led to an increase in LC3‐II conversion and Beclin1 level (Figure 6B,D), indicating a possible negative correlation between TRIM38 and autophagic process. Immunofluorescence (IF) analysis of LC3 demonstrated similar trends in both breast cancer cell lines (Figure 6E,F). To further validate our observations, we conducted autophagic flux assays using an adenovirus encoding mCherry‐GFP‐LC3, which is based on the principle of the quenching of GFP in acidic lysosomal environment.^[^ ^49^ ^]^ Specifically, red dots (mCherry dots) represent autolysosomes and are positively associated with autophagy flux activation. Yellow dots (GFP‐mCherry dots) represent autophagosomes, and an increase in the ratio of yellow dots/red dots indicates the obstruction of autophagic flux.^[^ ^50^ ^]^ Our results showed that TRIM38 overexpression reduced the number of red dots and increased the ratio of yellow dots to red dots, suggesting a blockade of autophagic flux in breast cancer cells (Figure 6G,H). In contrast, endogenous knockdown of TRIM38 enhanced autophagic flux (Figure 6G,H). Taken together, these data demonstrated that TRIM38 negatively regulated autophagic flux.

### 2.7 TRIM38‐Mediated Ubiquitination of SQSTM1 K420 Impedes Autophagic Flux by Blocking Its LIR Domain

**Figure 7:**
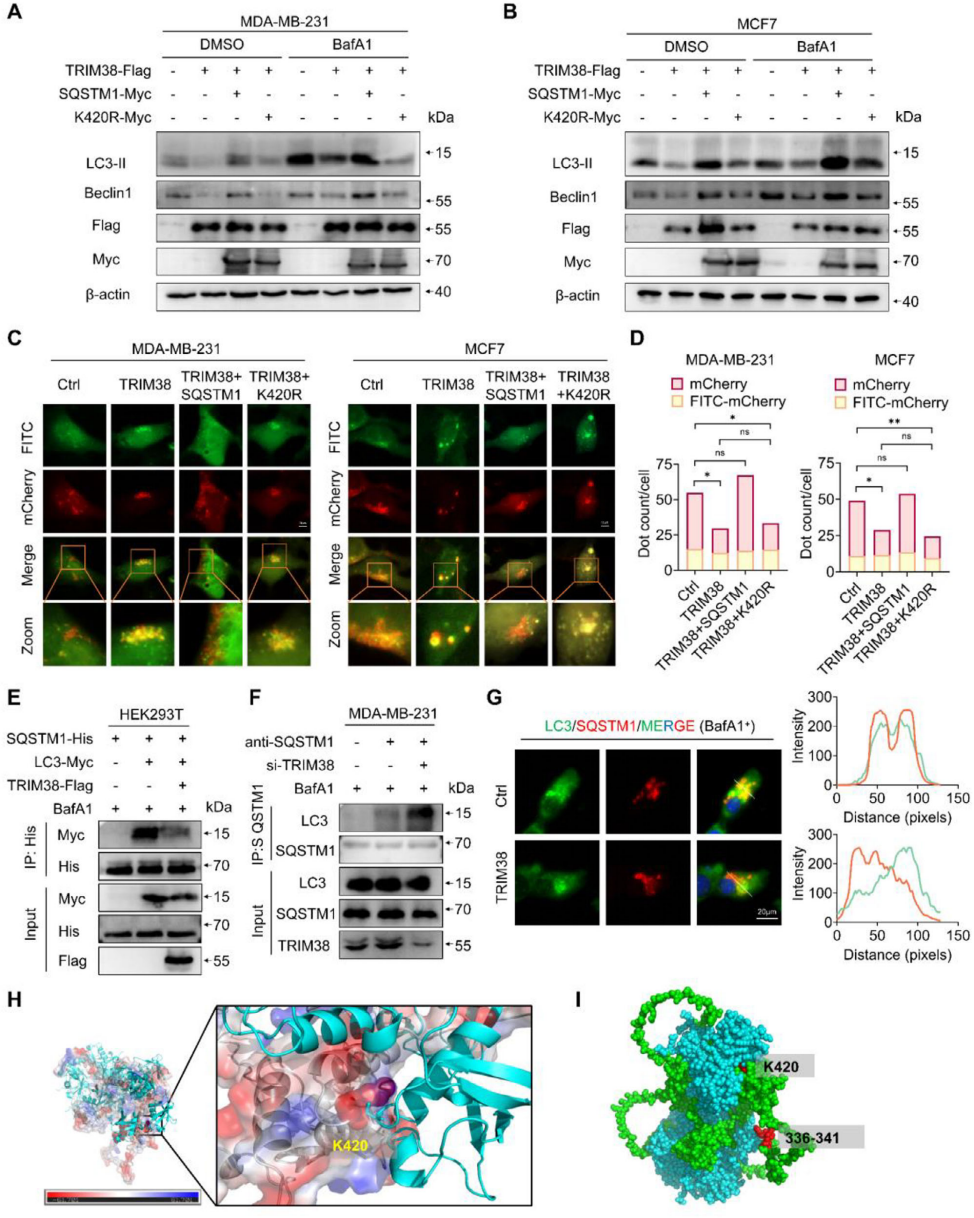
Ubiquitination of SQSTM1 K420 by TRIM38 may impede autophagic flux by blocking its LIR domain. A,B) Western blotting of lysates from MDA‐MB‐231 (A) and MCF7 (B) cells transfected with indicated plasmids. C,D) Autophagy flux assays were conducted in MDA‐MB‐231 and MCF7 cells transfected with the aforementioned plasmid combinations. E) HEK293T cells were co‐transfected with His‐SQSTM1, Flag‐TRIM38, and Myc‐LC3 for over 24 h. Following treatment with BafA1 for 6–8 h, lysates were immunoprecipitated using anti‐His and immunoblotted with anti‐Myc antibody. F) With or without TRIM38 knockdown, endogenous immunoprecipitation was performed in MDA‐MB‐231 cells after the treatment of BafA1 for 6 hours, using anti‐SQSTM1 antibody for immunoprecipitation. G) Immunofluorescence analysis of SQSTM1‐LC3 colocalization was performed in MDA‐MB‐231 cells with or without TRIM38 overexpression, following 6‐hour treatment with BafA1. H,I) Structural modeling of the complex formed between SQSTM1 K420 residue and a K63‐linked ubiquitin chain (represented as blue spheres). Significance was determined by unpaired two‐tailed Student's *t*‐test: *p < 0.05; **p < 0.01. All experiments were repeated at least three times and the results of representative experiments are shown.

To delve deeper into the mechanism by which TRIM38 regulates autophagic flux through ubiquitinating SQSTM1, we conducted rescue experiments. Western blot analysis (**Figure** **7**A,B) and autophagic flux assays (Figure 7C,D) consistently demonstrated that wild‐type SQSTM1, but not the ubiquitination‐defective mutant SQSTM1 K420R, could rescue the downregulation of autophagic flux induced by TRIM38. These findings supported our hypothesis regarding the functional impairment of SQSTM1 upon interaction with TRIM38. Given the established role of SQSTM1 in autophagy, we performed Co‐IP assays to investigate the interaction between SQSTM1 and LC3. Our results showed that TRIM38 decreased the binding between SQSTM1 and LC3 in HEK293T cells (Figure 7E). Correspondingly, knockdown of TRIM38 enhanced the endogenous interaction between SQSTM1 and LC3 in breast cancer cells (Figure 7F). Then IF analysis further revealed that TRIM38 disrupted the co‐localization of SQSTM1 and LC3 (Figure 7G). To gain insights into the structural basis of this interaction, we employed molecular docking to construct a virtual model of SQSTM1 ubiquitinated at K420 with a K63‐linked ubiquitin chain (Figure 7H). This model suggested that the K63 ubiquitin chain linked to K420 could sterically hinder the LIR domain (amino acids 335–341) of LC3, potentially leading to the separation of SQSTM1 and LC3 (Figure 7I). Collectively, these data indicated that ubiquitination of SQSTM1 K420, caused by TRIM38, impeded autophagic flux by disrupting the interaction between SQSTM1 and LC3 through its LIR domain blockage.

### 2.8 Tumor‐Suppressing Effect Mediated by TRIM38 Could be Rescued by SQSTM1 and Autophagy Agonist but not SQSTM1 K420R

**Figure 8:**
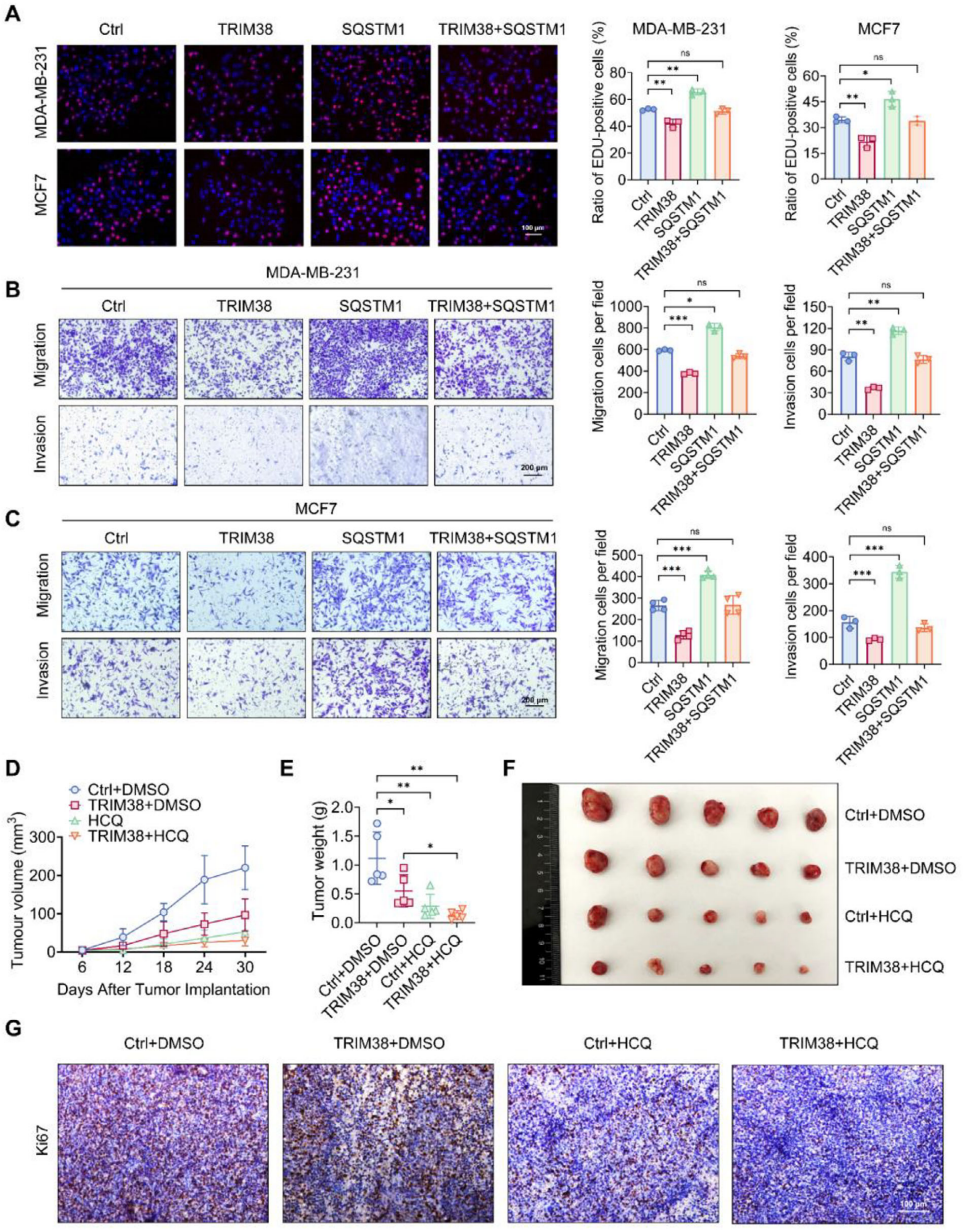
SQSTM1 can reverse the tumor‐suppressing effect of TRIM38. A) EdU assays for cell proliferation performed in MDA‐MB‐231 and MCF7 across experimental groups. B,C) Transwell assays across experimental groups in MDA‐MB‐231 cells (B) and MCF7 cells(C). D–F) MDA‐MB‐231 cells with or without endogenous TRIM38 overexpression was implanted on BALB/c‐nude (n = 5). Hydroxychloroquine (HCQ) or solvent was injected once every two days (i.p., 100 mg kg^−1^) in separate groups. Tumor growth curve (D), tumor weight (E), and tumor images (F) were as shown. G) Immunohistochemical staining for Ki67 among the indicated groups. Significance was determined by unpaired two‐tailed Student's *t*‐test: *p < 0.05; **p < 0.01; ***p < 0.001; ns, no significance. All experiments were repeated at least three times and the results of representative experiments are shown.

Based on the above results, we made the following conjecture: TRIM38‐mediated ubiquitination abrogates the interaction between SQSTM1 and LC3, thereby preventing SQSTM1 from entering lysosomes, which results in a blockade of autophagic flux and an accumulation of SQSTM1 due to reduced lysosomal degradation. To address whether TRIM38‐driven biological effects are mechanistically dependent on SQSTM1 dysregulation, we propose to test if restoring functional SQSTM1 could rescue the autophagy‐lysosomal impairment and reverse downstream cellular phenotypes. According to the above speculation, series rescue experiments were performed. In vitro cell proliferation assays, including MTT, EdU, and cell plate colony formation assays, revealed that breast cancer cells transfected with SQSTM1 exhibited a higher growth rate compared to the control group. The growth rate of cells overexpressing TRIM38 was slower than that of control cells, and cells co‐transfected with TRIM38 and SQSTM1 showed no significant difference from the control group (**Figure** **8**A; Figure S6A–C, Supporting Information). However, transfection of SQSTM1 K420R failed to reverse the growth inhibitory effect mediated by TRIM38 in vitro (Figure S6D,E, Supporting Information). Furthermore, results of transwell assays and wound healing assays suggested that migration and invasion abilities of breast cancer cells could be decreased upon being transfection with TRIM38 and increased with SQSTM1 transfection, and both of these effects could be counteracted by co‐transfection with SQSTM1 and TRIM38 (Figure 8C,D). Similar to the proliferation assays results, SQSTM1 K420R mutation failed to counteract TRIM38‐dependent inhibition of cell migration and invasion (Figure S6F–I, Supporting Information).

**Figure 9:**
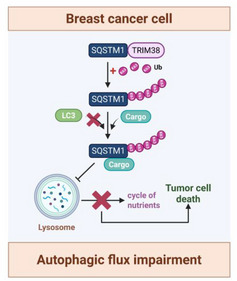
Schematic representation illustrating the mechanisms through which TRIM38 regulates the autophagic flux.

To further validate TRIM38 suppresses tumor progression through autophagy regulation, a commonly used autophagy agonist rapamycin (inhibition of the mTOR pathway), and an autophagy inhibitor hydroxychloroquine (HCQ), which is a chloroquine derivative currently in phase I/II clinical trials for various tumors,^[^ ^51^ ^]^ were employed for rescue experiments. Our results demonstrated that rapamycin could almost rescue the suppressive effect induced by TRIM38 on breast cancer cell proliferation (Figure S7A,B, Supporting Information), migration, and invasion (Figure S7C–F, Supporting Information). Conversely, when treated with HCQ, cells with endogenous knockdown of TRIM38 showed no significant decrease in proliferative capacity (Figure S8A,B, Supporting Information), migrative capacity, and invasive capacity (Figure S8C–F, Supporting Information), suggesting that HCQ could offset the enhancing effect on cancer progression induced by the absence of TRIM38. Subsequently, we conducted a tumor implantations experiment using TRIM38‐overexpressed MDA‐MB‐231 in combination with HCQ. The results showed that high‐level expression of TRIM38 could enhance the proliferation‐suppressing effect of HCQ in vivo (Figure 8D–F). Ki67 staining also decreased significantly in the combination group (Figure 8G). These findings demonstrate that TRIM38 inhibits tumor progression by mediating ubiquitination‐induced SQSTM1 dysfunction during autophagy, highlighting the therapeutic potential of pharmacological TRIM38 activation combined with autophagy inhibitors. The simplified mechanism diagram is presented in (**Figure** **9**)

## 3 Discussion

Breast cancer ranks among the three most prevalent cancers globally, alongside lung cancer and colon cancer, and has surpassed others to become the second leading cause of cancer‐related deaths.^[^ ^52^, ^53^ ^]^ In recent years, advancements in genomic research, pathological techniques, and imaging analysis have significantly transformed our understanding of the molecular mechanisms underlying the initiation and progression of breast malignancy.^[^ ^54^ ^]^ The elucidation of pivotal molecules and their regulatory pathways is crucial for identifying therapeutic targets and prognostic biomarkers that can effectively address breast cancer. In this study, we identified TRIM38 as a novel biomarker associated with low malignancy, exhibiting elevated expression in adjacent non‐cancerous tissues compared to tumor tissues. Furthermore, our results indicated that elevated TRIM38 expression was significantly associated with improved survival, including OS, DDFS, RFS, and BCRFS, establishing its prognostic value in breast cancer patients.

The dual role of autophagy in tumor progression underscores the therapeutic potential of targeting its key regulatory molecules, which could yield novel treatment strategies. Members of the TRIM family have been reported to regulate various autophagic machinery through different strategies, functioning as scaffold proteins or via ubiquitin‐mediated mechanisms.^[^ ^55^ ^]^ TRIM38, a member of TRIM family with a unique PRY/SPRY domain, has been recognized as an essential member in regulating innate immune and inflammatory responses previously.^[^ ^5^ ^]^ However, no evidence has yet indicated that TRIM38 is involved in the regulation of autophagy processes. Our study is the first to uncover the crucial role of TRIM38 in blocking autophagic flux by disrupting the late stages of autophagy. TRIM38‐mediated ubiquitination of SQSTM1 prevents its binding to LC3 on lysosomal membranes, leading to impairment of ubiquitinated substrate degradation and obstruction of intracellular nutrient recycling.

Additionally, our findings highlight the translational potential of combining TRIM38 agonists with autophagy inhibitors (e.g., HCQ). The majority of tumors are diagnosed at an advanced or metastatic stage, prompting therapeutic strategies that target autophagic processes to primarily focus on autophagy inhibition. Currently, chloroquine derivatives, particularly HCQ, are the sole autophagy inhibitors available for clinical use. Following their long history of application in malaria and rheumatism, these drugs have been reintroduced into numerous clinical trials for treating various cancers in recent years.^[^ ^56^ ^]^ However, the efficacy and pharmacological properties of HCQ may be limited by various factors. Consequently, combination therapy may offer an opportunity to enhance the antitumor effect of HCQ. Our findings suggest that endogenous high expression of TRIM38 can potentiate the tumor growth inhibition mediated by HCQ, exhibiting a synergistic antitumor effect in mouse model.

Furthermore, building upon our mechanistic characterization of TRIM38/SQSTM1 axis, we proposed two possible intervention strategies for future clinical therapies. First, small‐molecule targeted drugs offer advantages including superior pharmacokinetic, cost‐effectiveness, enhanced patient compliance, and streamlined pharmaceutical logistics.^[^ ^57^ ^]^ Screening existing small‐molecule libraries could readily identify TRIM38 agonists. Second, proteolysis‐targeting chimeras (PROTACs) constitute heterobifunctional molecules with two distinct ligand moieties connected by a chemical linker: one binding the target protein and the other recruiting an E3 ubiquitin ligase to enable ubiquitin‐mediated proteasomal degradation.^[^ ^58^ ^]^ Applying this paradigm, we envision a chimera comprising a TRIM38 ligand, a chemical linker, and an SQSTM1 ligand to trigger TRIM38/SQSTM1‐mediated autophagic obstruction. Both approaches outline pharmacologically actionable pathways to translate autophagy modulation mechanisms into clinical cancer therapeutics.

Notably, our study presents some intriguing results that warrant further investigation. Prior investigations have elucidated the intricate regulatory roles of SQSTM1 K420 ubiquitination in modulating its abundance and functional attributes. Specifically, it has been documented that SCOP‐facilitated K27‐ and K29‐linked ubiquitination of SQSTM1 K420 diminishes its ability to form puncta, undergo liquid‐phase condensation, and bind ubiquitin, consequently inhibiting SQSTM1‐mediated autophagy.^[^ ^59^ ^]^ Additionally, another study has reported that USP8 can directly cleave K11‐linked ubiquitin chains at SQSTM1 K420, thereby suppressing its autophagic activity.^[^ ^37^ ^]^ In our study, we uncovered that K63‐linked ubiquitination at SQSTM1 K420 similarly impairs its function and attenuates autophagy flux. These observations collectively highlight that diverse ubiquitination modifications at the same residue can exert distinct effects on the functional properties of the substrate protein and associated pathways.

TRAF2 has also been reported to promote K63‐linked ubiquitination at SQSTM1 K420, resulting in the formation of the SQSTM1‐mTORC1‐Rag complex and autophagy suppressing.^[^ ^48^ ^]^ However, in this study, researchers have found that TRAF2 negatively regulates the protein levels of SQSTM1 through degradation in concert with mTORC1, which contradicts our finding of an increased SQSTM1 protein level following K63‐ubiquitination. Why does the same modification at the same site lead to different outcomes? We propose two probable reasons for this disparity. Firstly, previous reports have indicated that the length of the ubiquitin chain significantly influences its ability to be selectively recognized by ubiquitin‐binding proteins.^[^ ^60^ ^]^ Different ubiquitinating enzymes may attach ubiquitin chains of varying lengths to SQSTM1, thereby causing different functional alterations. Secondly, the binding modalities between ubiquitin chains and substrate proteins encompass covalent and non‐covalent binding, which represents a dynamic quaternary structure formed between two adjacent ubiquitin monomers.^[^ ^61^ ^]^ Such interactions enable the subunits to form different conformations. For instance, K63‐linked dimeric ubiquitin (K63‐diUb) can exist in two interconversion conformations: open or compact,^[^ ^62^ ^]^ binding different target proteins and mediating distinct functions.^[^ ^63^, ^64^ ^]^ The IP assays, employed in the aforementioned study, demonstrated the amount of both covalently and non‐covalent bound ubiquitin. In our study, we utilized a two‐step IP, which only revealed covalently bound ubiquitin,^[^ ^34^ ^]^ to identify the modification site on SQSTM1. The different effects of TRAF2 and TRIM38 on SQSTM1 may be attributed to the different ubiquitin linkage patterns. However, due to the lack of in‐depth investigation of the corresponding ubiquitin chains in both studies, we are unable to objectively determine the specific structure of the ubiquitin chain added by either molecule. We also anticipate crystallographic explanations to resolve this contradiction in the future.

While our data provide evidence for autophagy regulation mechanism involving TRIM38, several limitations need to be acknowledged. First, due to technical constraints, this study did not explore potential TRIM38 agonists. Thus, we eagerly anticipate the development of TRIM38 agonists, which hold potential application prospects in combination with autophagy inhibitors and may improve antitumor treatment. Additionally, although our findings delineate a new ubiquitination‐dependent axis of autophagy regulation in breast cancer, the spatiotemporal control of this pathway in different tumor contexts warrants further exploration. Finally, the limitation of a relatively small clinical sample size should be acknowledged. This led to wider confidence intervals and decreased precision in estimating effect sizes, indicating that the results should be interpreted with caution due to the broader range of plausible population values. Nonetheless, the internal consistency of the findings across multiple analytical approaches and their concordance with the proposed biological mechanism enhance the credibility of the observations. This coherence suggests that the effects detected are robust within the present cohort and justifies further investigation through larger, sufficiently powered studies to more accurately quantify the effects and validate the generalizability of our conclusions.

Collectively, our work demonstrates that TRIM38 functions as a tumor suppressor in breast cancer progression and establishes its potential as a prognostically favorable biomarker associated with prolonged survival in breast cancer patients. Mechanistically, TRIM38 mediates non‐degradative K63‐linked ubiquitination of SQSTM1, which reduces its binding with LC3, leading to autophagic flux blockade and nutrient recycling disruption. These findings deepen our understanding of autophagy regulation and lay the groundwork for developing specific agonists to regulate this process in cancer, which is of great significance for cancer treatment.

## 4 Experimental Section

### Cell Culture

MDA‐MB‐231(RRID: CVCL_0062), MCF7(RRID: CVCL_0031), and HEK293T (RRID: CVCL_0063) cells were obtained from the American Type Culture Collection (Manassas, VA, USA). All cell lines were maintained at 37 °C in a humidified incubator with 5% CO_2_, atmosphere, using Dulbecco's Modified Eagle Medium (DMEM) supplemented 10% fetal bovine serum (FBS), 100 U mL^−1^ penicillin, and 100 µg mL^−1^ streptomycin. And the study confirms the cell line was contamination‐free.

### Plasmids and Transfection

Expression plasmids, including HA‐tagged ubiquitin variants (WT, K6, K11, K27, K29, K33, K48, K63), were kindly gifted by Professor W. Zhao of Shandong University, Jinan, China. His‐tagged SQSTM1 (P48811), Myc‐tagged SQSTM1 (P1815), Myc‐tagged MAP1LC3B (P66141), Myc‐tagged USP8 (P22482), and Myc‐tagged CYLD (P23119) were obtained from MiaoLingBio, China. Flag‐tagged human TRIM38 was synthesized via PCR amplification of cDNA isolated from HEK293T cells and cloned into the pCMV6‐Entry mammalian expression vector (PS100001) sourced from OriGene Technologies (Wuxi, China), with the Myc tag silenced using the KOD‐Plus‐Mutagenesis kit (Toyobo, Osaka, Japan). This kit was also utilized to generate the TRIM38 C16A mutant and truncated isoforms of TRIM38 and SQSTM1. All constructs underwent comprehensive DNA sequencing to confirm their authenticity. For functional studies, MDA‐MB‐231, MCF7, and HEK293T cells were transfected with the selected plasmids or si‐RNAs using jetPRIME reagent (Polyplus Transfection, France), according to the manufacturer's instructions.

### RT‐PCR

Total RNA was extracted using TRIzol reagent (Thermo Fisher Scientific, USA), following the manufacturer's protocol. Subsequently, 500 ng of RNA was reverse‐transcribed using Evo M‐MLV RT Premix for qPCR (ACCURATE BIOTECHNOLOGY(HUNAN) CO., LTD, ChangSha, China). RT‐PCR analysis was performed using the LightCycler 480 Instrument (Roche Diagnostics, Basel, Switzerland) and Hieff qPCR SYBR Green Master Mix (No Rox) (11201ES) from Yeasen Biotechnology (Shanghai, China). The expression data for each sample were normalized to β‐actin expression. The specific primers used for real‐time PCR are listed in Table S1 (Supporting Information).

### Immunoprecipitation (IP) and Western Blotting

24 hours post‐transfection, cells were lysed using lysis buffer (Beyotime, P0013) containing PMSF and NaF on ice for 30 min. The lysate was then centrifuged at 12000×rpm for 20 min at 4 °C. Subsequently, the supernatants were incubated overnight at 4 °C with protein A/G Plus‐Agarose (Santa Cruz Biotechnology) and specific antibodies in a mixing apparatus. The beads were washed five times with IP buffer containing NP‐40 buffer (Beyotime) and boiled in 1× SDS loading buffer to elute proteins. Prepared IP samples or fresh cell lysates were then separated by SDS‐PAGE and transferred onto PVDF membranes, which were blocked with 5% bovine serum albumin (BSA) for 1 h. Blots were incubated overnight with primary antibodies at 4 °C, followed by a 1‐hour incubation with secondary HRP‐conjugated antibodies. Protein bands were visualized using an ECL detection system (WBKLS0500) (Merck Millipore).

### Re‐IP Assay

HEK‐293T cells were co‐transfected with Myc‐tagged SQSTM1, HA‐ubiquitin, and Flag‐TRIM38. Cell lysates underwent initial immunoprecipitation with anti‐Myc antibody, whereafter the immunoprecipitates were denatured (boiled in 1%SDS) and the supernatant was subjected to sequential re‐immunoprecipitation (Re‐IP) with Myc antibody in IP buffer again. The immunoprecipitates were then subjected to western blot. This protocol enhances specificity by reducing non‐specific binding and background noise, and has been widely employed for detecting ubiquitination.^[^ ^65^ ^]^

### EdU Assay

The EdU cell proliferation assay was conducted using the EdU detection kit (C10310‐1) (RiboBio, Guangzhou, China), following the manufacturer's instructions. Cells cultured in 96‐well plates were incubated with 100 µL 5‐ethynyl‐2′‐deoxyuridine diluent at 37 °C for 2 h. The cells were then fixed with 4% paraformaldehyde for 20 min, permeabilized with 0.4% Triton X‐100 for 10 min, and incubated with 100 µL of Apollo reaction cocktail for 30 min. After staining with Hoechst for 30 min, representative images were captured under a Nikon inverted fluorescence microscope. The cell proliferation rate was determined by calculating the ratio of EdU‐positive cells (red) to Hoechst‐positive cells (blue).

### Cell Plate Colony Formation Assay

1 × 10^3^ tumor cells in logarithmic growth phase were seeded into 6‐cm petri dishes and cultivated at 37 °C in a 5% CO_2_ atmosphere. Fresh medium was replaced every 3 days. After an incubation period of approximately 14–21 days, colonies were fixed with methanol, stained with crystal violet, and counted using Image J software.

### Transwell Assay

Following a 6‐hour starvation period, transfected or treated cells were plated into the upper chamber of a transwell assay system at a density of 8 × 10^4^ cells per well. For invasion assays, the upper chamber of the transwell filter was pre‐coated with Matrigel (Corning Incorporated, Corning, NY, USA), whereas it remained uncoated for migration assays. Media supplemented with 20% FBS was added to the lower chamber, and cells were incubated for 24–36 h. Non‐migratory/non‐invasive cells in the upper chamber were gently removed using a swab. Cells that migrated or invaded into the lower chamber were fixed in methanol for 10 minutes, stained with 0.1% crystal violet for 15 minutes, and then imaged using a Nikon inverted microscope. Migration and invasion abilities were quantified through transwell migration assays (uncoated inserts) and transwell invasion assays (Matrigel‐coated inserts), respectively.

### MTT Assay

To perform the MTT assay, 1 × 10^3^ cells were plated into 96‐well plants in triplicate. The MTT reagent, 3‐(4,5‐dimethylthiazol‐2‐yl)‐2,5‐diphenyl‐2H‐tetrazolium bromide, was prepared at a concentration of 5 mg mL^−1^ in water. 50 µL of MTT reagent was added to each well containing cells, while a control well received 50 µL of MTT reagent mixed with 50 µL of cell culture media (devoid of cells). The plate was incubated at 37 °C for 4 hours. Then the supernatant was aspirated, and 100 µL of MTT solvent (DMSO) was added to each well. The plate was wrapped in aluminum foil and shaken on an orbital shaker for 5 minutes. Absorbance was measured at OD = 490 nm. For data analysis, the background absorbance of the cell culture medium was subtracted from the experimental readings, and the duplicate readings for each sample were averaged.

### Wound Healing Assay

For the wound healing assay, transfected or treated cells were plated in 24‐well plates and cultured to 90% confluence. A standardized wound was generated in the monolayer using a sterile 10 µL pipette tip. After five washes with 1× PBS, cells were incubated in serum‐free medium for 24 h. Wound closure was evaluated at 0 and 24 h using a Nikon inverted microscope. The width of the wound is determined by “Image J” software. The formula for calculating the percentage of wound closure is as follows: Wound closure = [(Width_0h_‐ Width_24h_)/ Width_0h_] × 100%

### Liquid Chromatography Tandem Mass Spectrometry (LC‐MS/MS)

Immunoprecipitation (IP) was performed first using an anti‐TRIM38 antibody (rabbit IgG as isotype control) with whole‐cell lysates from MDA‐MB‐231 cells. Immunoprecipitated proteins were resolved by SDS‐PAGE and stained with Coomassie Brilliant Blue staining. After confirming the IP efficiency via western blotting, gel regions corresponding to specific bands were excised. All subsequent LC‐MS/MS procedures, including sample preparation, chromatographic separation, and mass spectrometric analysis, were conducted by Qinglian Bio (Beijing, China). Raw data processing, peptide identification, and bioinformatic analysis were conducted by Qinglian Bio using the Uniprot human database.

### Animal Experiments

To establish subcutaneous tumor‐bearing models, Balb/c‐nude mice were injected subcutaneously in the right flank with 5 × 10^6^ cells in 100 µL PBS. Tumor growth was monitored every 6 days from day 6 post‐injection by measuring the longest (L) and shortest (S) diameters using vernier calipers. Tumor volume(V) was calculated as V = (L × S^2^)/2. Mice were euthanized by CO_2_ asphyxiation when tumors reached ≥15 mm in diameter or at day 40 post‐inoculation, whichever occurred first. Tumors were then harvested for necropsy and downstream analysis. Treatment groups received intraperitoneal injections (100 µL) of either HCQ (100 mg kg^−1^) dissolved in sterile vehicle (4% NMP, 3% Tween‐80, 20% PEG400 in Milli‐Q water) or vehicle control every other day for 21 days. Body weight and clinical signs were recorded daily to assess treatment tolerability.

### H&E Staining

H&E staining for tumor tissues was conducted using the HD‐400i Automatic Dyeing Machine (Chiwell Bio, Ningbo).

### Immunohistochemistry

Immunohistochemical (IHC) analysis was performed on paraffin‐embedded tissue samples according to a previously established protocol.^[^ ^66^ ^]^ Antigen retrieval was achieved using Tris‐EDTA antigen retrieval solution (PR30002, Proteintech). Pathological sections were incubated with primary antibodies targeting Ki67 (1:1000) and TRIM38 (1:200) at 4 °C overnight. Secondary antibody incubation was conducted as per the instructions provided in the IHC reagent kit (PV 9000, ZSGB‐BIO). Diaminobenzidine (DAB) (ZLI‐9018, ZSGB‐BIO) served as the chromogen, and hematoxylin was used for counterstaining.

### Semi‐Quantitative Evaluation for IHC Score

Immunohistochemical staining was evaluated using light microscopy. Initial screening was performed at 100× magnification (10×objective) to identify tumor tissues, followed by detailed analysis at 400×magnification (40×objective) to assess staining characteristics. Staining intensity (***i***) was graded on a 0 to 3 scale: 0 (negative), 1 (weak), 2 (moderate), and 3 (strong). The percentage of cells at each intensity level (***Pi***) was recorded from 0% to 100%. The IHC score was calculated using the formula: IHC score = Σ (***i*** × ***Pi***), yielding a theoretical range of 0–300.^[^ ^67^, ^68^ ^]^ Two experienced pathologists independently evaluated TRIM38 staining of breast carcinoma regions (including both in situ and invasive components) and assigned IHC scores. The average of their scores was used as the final IHC score for each patient. Patients were stratified into two groups based on the cohort's median IHC score, which is 140 in this research: TRIM38‐high expression group (above median) and TRIM38‐low expression group (below median). A slight imbalance in group size resulted from the application of the median cut‐off to a limited sample set where several cases had expression levels at the median value.

### Tissue Protein Extraction

Tissue specimens were minced into fragments of ≈2 mm^3^, transferred to pre‐chilled 1.5 mL microcentrifuge tubes, and combined with two stainless steel grinding beads and 500 µl RIPA lysis buffer containing protease inhibitor cocktail and PMSF. Homogenization was performed using a cryogenic grinder (model JXFSTPRP‐CLN‐48), followed by 2‐hour lysis on ice. Lysates were centrifuged at 15 000 rpm for 30 min at 4 °C, with the supernatant collected as tissue protein lysate.

### Immunofluorescence Staining and Confocal Analysis

MDA‐MB‐231 and MCF7 cells were seeded onto glass coverslips within 24‐well plates or confocal dishes. Subsequently, the cells were fixed in methanol for 15 min at room temperature. After fixation, they were blocked with 10% BSA in PBS for 1 h, and then were incubated overnight at 4 °C with primary antibodies (TRIM38, SQSTM1, LC3). Following three washes with PBST, secondary antibodies (FITC‐ or Rhodamine‐labeled IgG) and DAPI were applied in sequence to the coverslips or confocal dishes and incubated for 30 min at room temperature. Finally, the cells were analyzed using the Zeiss LSM980 confocal microscope available at Shandong University's Translational Medicine Sharing Platform.

### Molecular Modeling and Protein‐Protein Docking

The amino acid sequences of TRIM38 (O00635) and SQSTM1 (Q13501) obtained from UniProt were used for homology modeling via AlphaFold3. The structures of the complex were predicted by GRAMM docking. Subsequently, the docking results were visualized through Pymol 2.4.

### Autophagic Flux Assay

Autophagic flux was assessed using a tandem fluorescent‐tagged LC3 adenovirus (Beyotime, C3012) which exploits the different pH stabilities of mCherry and GFP. Breast cancer cells, post‐transfection, were infected with mCherry‐GFP‐LC3 adenovirus for 48–96 h. Zessi axio observer 7 was used to observe the cells. Yellow dots (co‐localization of mCherry and FITC signals) represented autophagosomes, while red dots (mCherry signal only) represented autolysosomes.

### Patient Samples

Tissue specimens were collected from breast cancer patients who underwent surgery and were histologically diagnosed in Qilu Hospital, Shandong University (Shandong, China). Comprehensive follow‐up records were maintained for all participants who provided written informed consent. This study was in accordance with the Helsinki Declaration and had obtained ethical approval from the Scientific Research Ethics Committee of Qilu Hospital, Shandong University.

### Statistical Analysis

All experiments were independently replicated at least three times. Results are presented as mean ± standard deviation (SD) with individual data points shown in scatter plots. Sample sizes (n) representing biological replicates are explicitly indicated in all figure captions and statistical comparisons. Statistical analyses were carried out using GraphPad Prism 10.1.2 and IBM SPSS 25.0. Inter‐group differences were evaluated by two‐tailed Student's *t*‐tests and one‐way ANOVA. Kaplan–Meier curves and log‐rank tests were used to compare patient survival outcomes. Univariate and multivariate Cox proportional hazards models were employed to identify independent prognostic factors. Results are presented as mean ± SD based on three experiments. Statistical significance was defined as *p* < 0.05.

### Ethics Approval and Consent to Participate

This study has been approved by the Scientific Research Ethics Committee of Qilu Hospital, Shandong University (KYLL‐2021(KS)‐674). All human pathology samples used in this study were obtained from Qilu Hospital, Shandong University, with all participants providing informed consent and ensuring strict privacy protection. Animal experiments complied with China's Guidelines for Animal Health and Use and were approved by the Ethics Committee of Qilu Hospital of Shandong University (DWLL‐202500307).

## Conflict of Interest

The authors declare no conflict of interest.

## Author Contributions

S.J. and L.W. contributed equally to this work. Q.Y. designed research studies. S.J. and L.W. performed most of the experiments of this work. P.S. collected patient samples. T.C. and D.L. analyzed data. B.C., W.Z., N.Z., X.W., Y.L., and Y.L. provided valuable discussion. L.W. and S.J. wrote the manuscript.

## Supporting information



Supporting Information

## Data Availability

The data that support the findings of this study are available from the corresponding author upon reasonable request.
