# Supplementary material for: TRIM38 Suppresses Breast Cancer Progression via Modulating SQSTM1 Ubiquitination and Autophagic Flux
Source: Adv Sci (Weinh). 2025 Dec 5;13(11):e12725. doi: 10.1002/advs.202512725 (PMC12931206; doi:10.1002/advs.202512725)
Supplement: Supplementary file 1 — Supporting Information [file ADVS-13-e12725-s001.docx]

Supplementary Materials for

**TRIM38 suppresses breast cancer progression via modulating SQSTM1 ubiquitination and autophagic flux**

Shan Jiang^1,^ ^†^, Lijuan Wang^2,^ ^†^, Dianwen Han^1^, Peng Su^3^, Bing Chen^2^, Wenjing Zhao^2^, Tong Chen^1^, Ning Zhang^1^, Xiaolong Wang^1^, Yiran Liang^1^, Yaming Li^1^, Chen Li^1^, Xi Chen^1^，Dan Luo^1^, Qifeng Yang^1, 2, 4, *^

^1^ Department of Breast Surgery, General Surgery, Qilu Hospital of Shandong University

^2^ Biological Resource Center, Qilu Hospital of Shandong University

^3^ Department of Pathology, Qilu Hospital of Shandong University

^4^ Research Institute of Breast Cancer, Shandong University.

†These authors contributed equally to this work.

^*^**Address correspondence to:** Qifeng Yang ([qifengy_sdu@163.com](mailto:qifengy_sdu@163.com)).

**SUPPLEMENTARY MATERIALS**

**Supplementary Figures and Figure Legends**

**
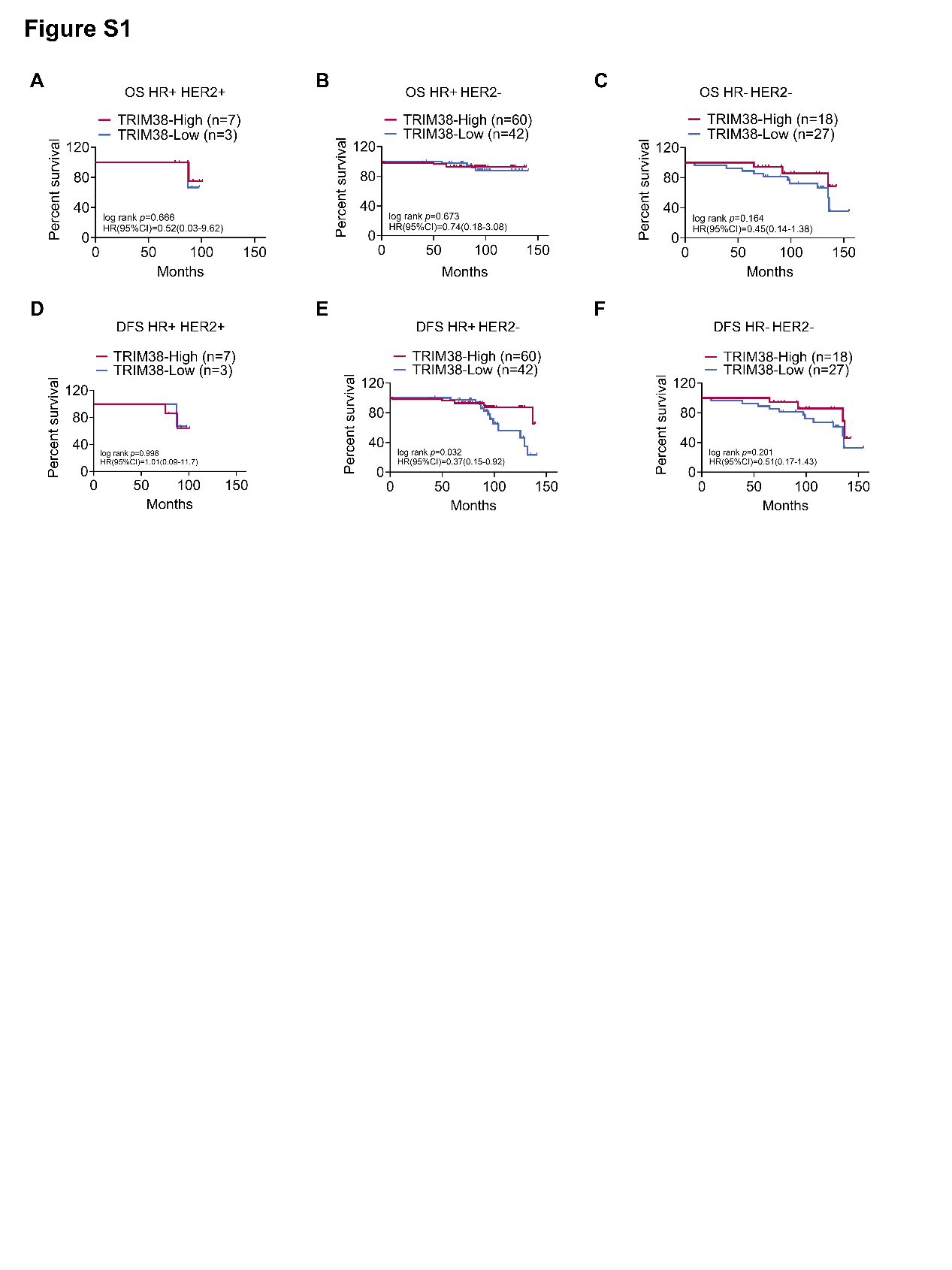
**

**Supplementary Figure 1.** **Survival analyses of TRIM38 expression in different subtypes of breast cancer patients.** Based on the immunohistochemical scores, overall survival (**A-C**) and disease-free survival (**D-F**) were analyzed in patients with high TRIM38 expression versus patients with low TRIM38 expression. Survival differences were assessed using Kaplan-Meier curves with log-rank tests.


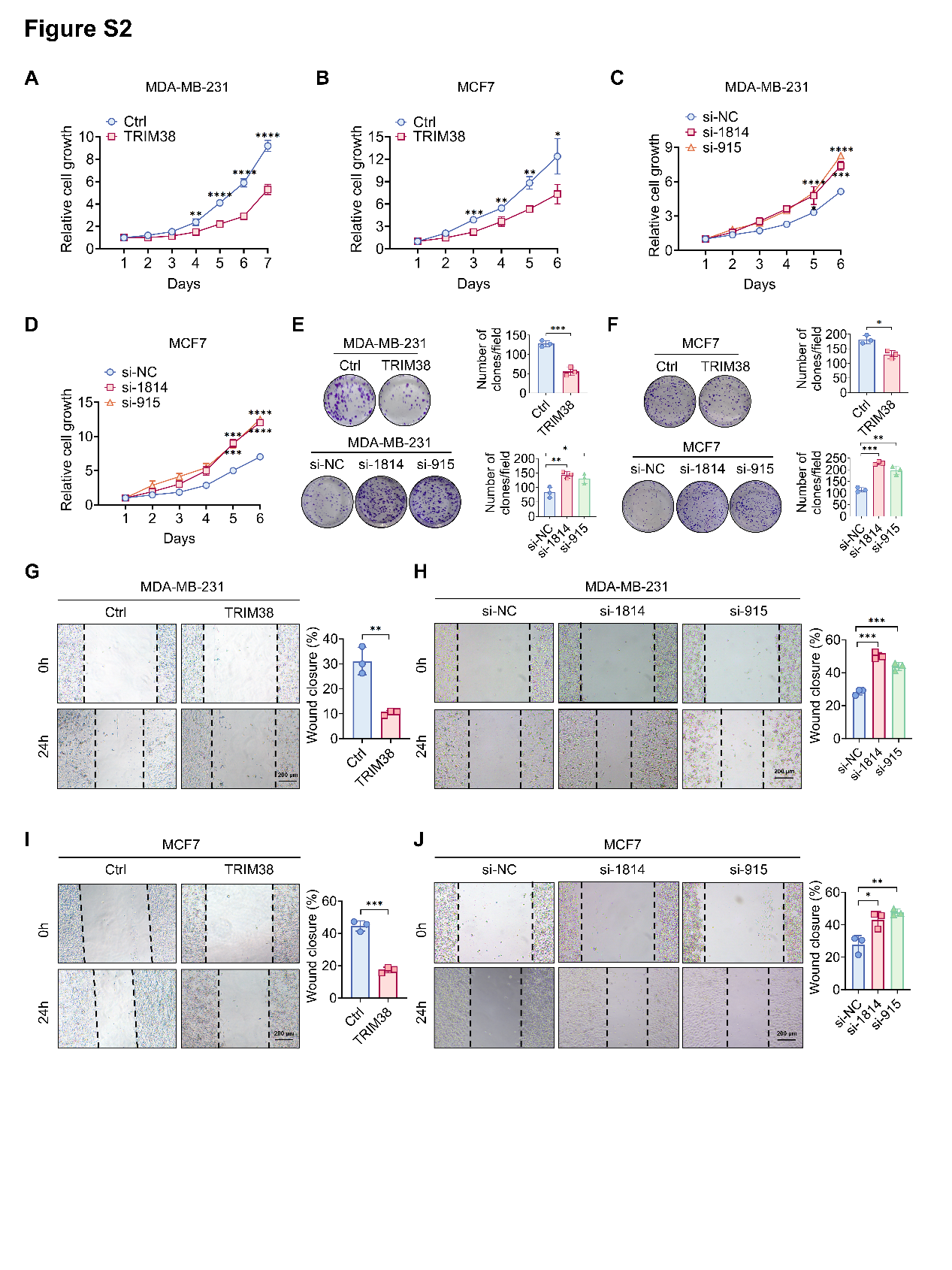


**Supplementary Figure 2. TRIM38 suppress breast cancer proliferation *in vitro*. A-B.** MTT assay to examine the effect of TRIM38 overexpression on the proliferation of MDA-MB-231 (**A**) and MCF7 (**B**) cells *in vitro*. **C-D.** MTT assay to examine the effect of TRIM38 knockdown on the proliferation of MDA-MB-231 (**C**) and MCF7 (**D**) cells *in vitro*. **E-F.** Cell plate colony formation assays using MDA-MB-231 **(E)** or MCF7 **(F)** transfected with TRIM38 plasmid or si-TRIM38. **G-H.** Wound healing assays for MDA-MB-231 transfected with TRIM38 plasmid (**G**) or si-TRIM38(**H**). **I-J.** Wound healing assays for MCF7 transfected with TRIM38 plasmid (**I**) or si-TRIM38 (**J**). (mean ±SD, two-tailed t-test, *p < 0.05; **p < 0.01; ***p < 0.001; ****p<0.0001). All experiments were repeated at least three times.

 
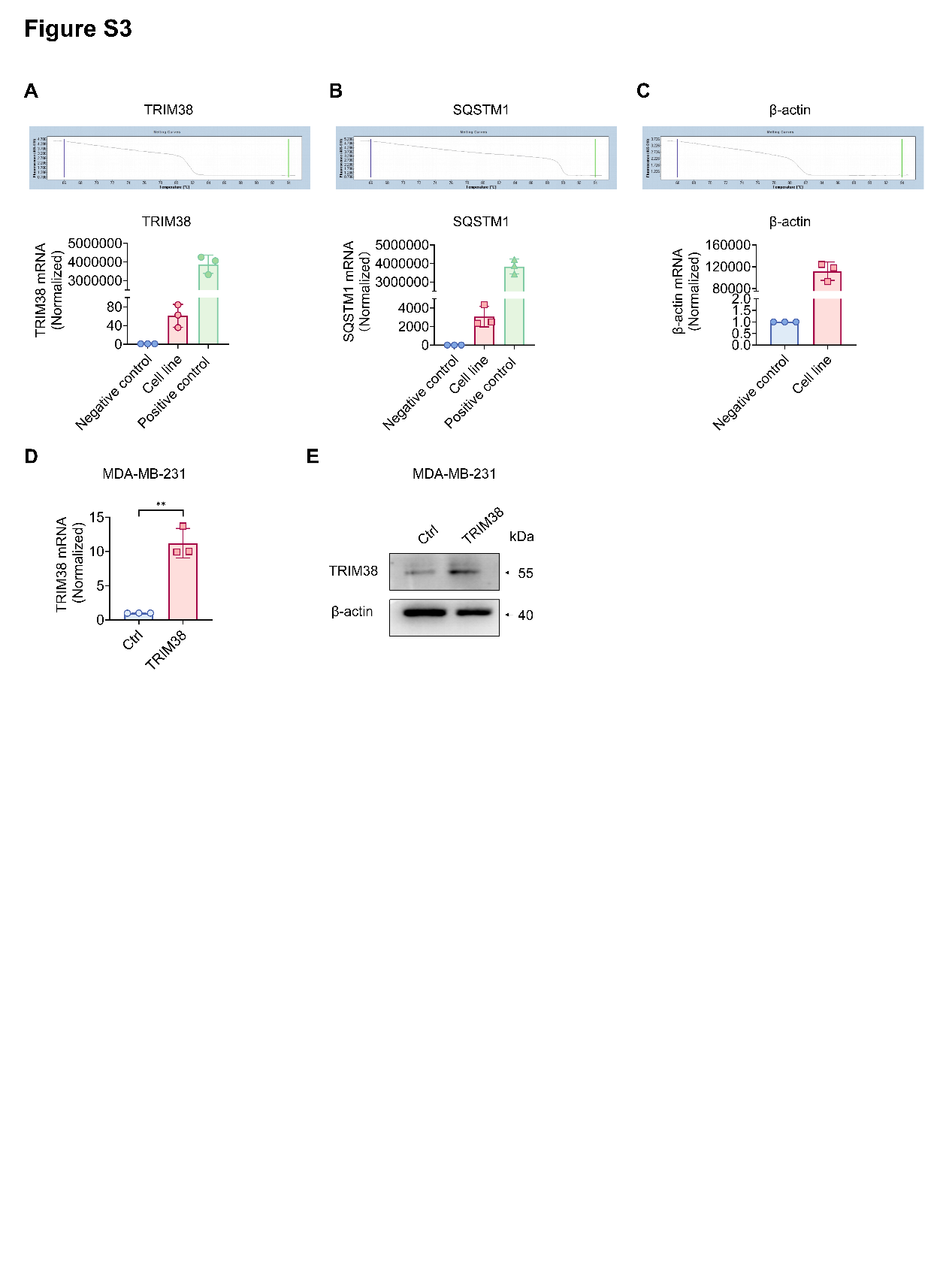


**Supplementary Figure 3. Validation of TRIM38 stable overexpression efficiency in cell lines. A-C.** Melting curves and negative (water)/positive (plasmid) controls for RT-qPCR primers used in this study. **D-E**. Examinations for overexpression efficiency of TRIM38-stable-overexpress MDA-MB-231 cells by RT-qPCR **(D)** and western blotting **(E).**

**
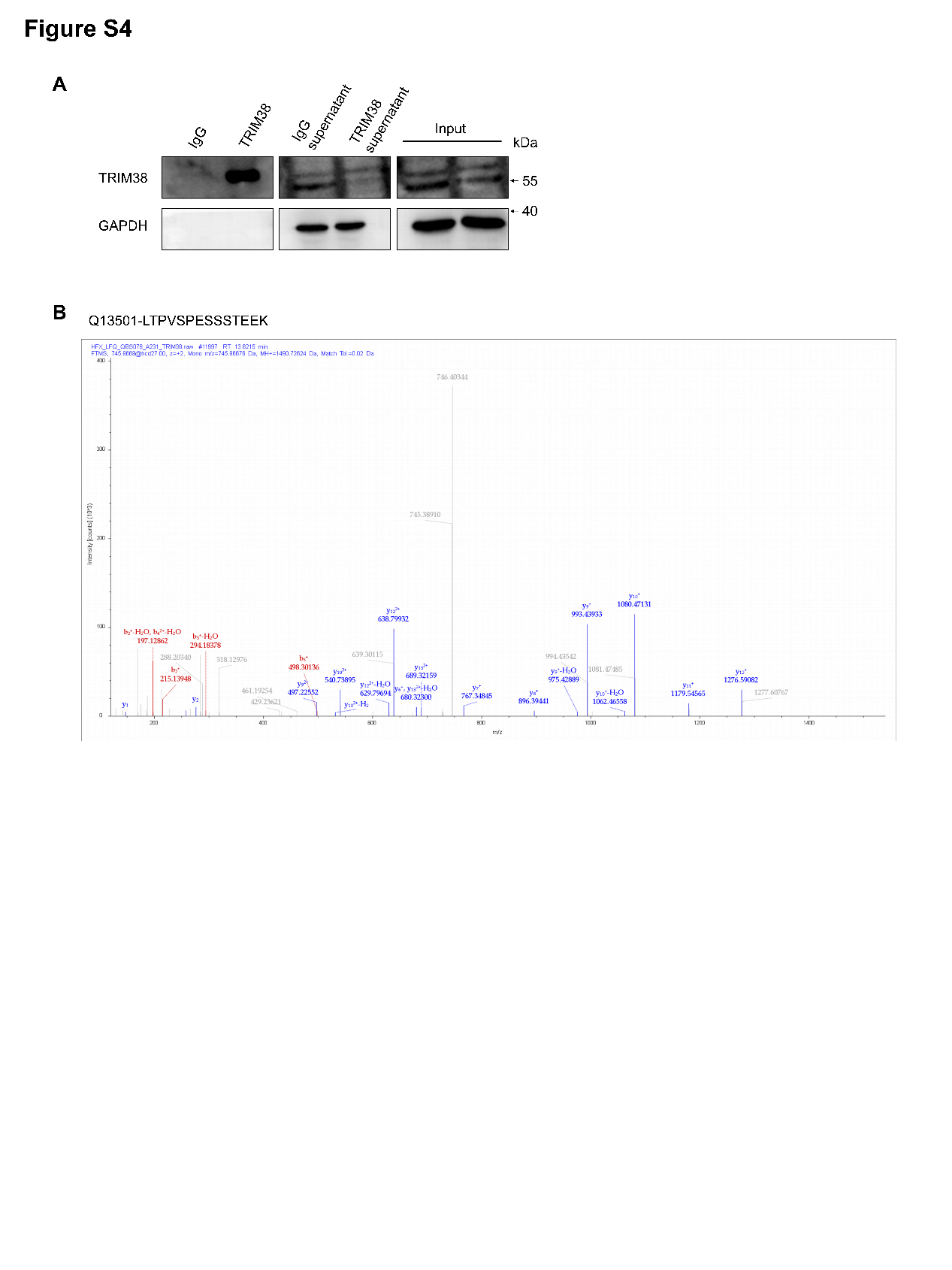
**

**Supplementary Figure 4. TRIM38 interacts with SQSTM1. A.** Western blot analysis to assess the efficiency of immunoprecipitation using anti-TRIM38 in MDA-MB-231 cell lysates. **B**. LC-MS/MS analysis reveals the protein SQSTM1 precipitated with anti-TRIM38 antibodies from the MDA-MB-231 cell lysates.

**
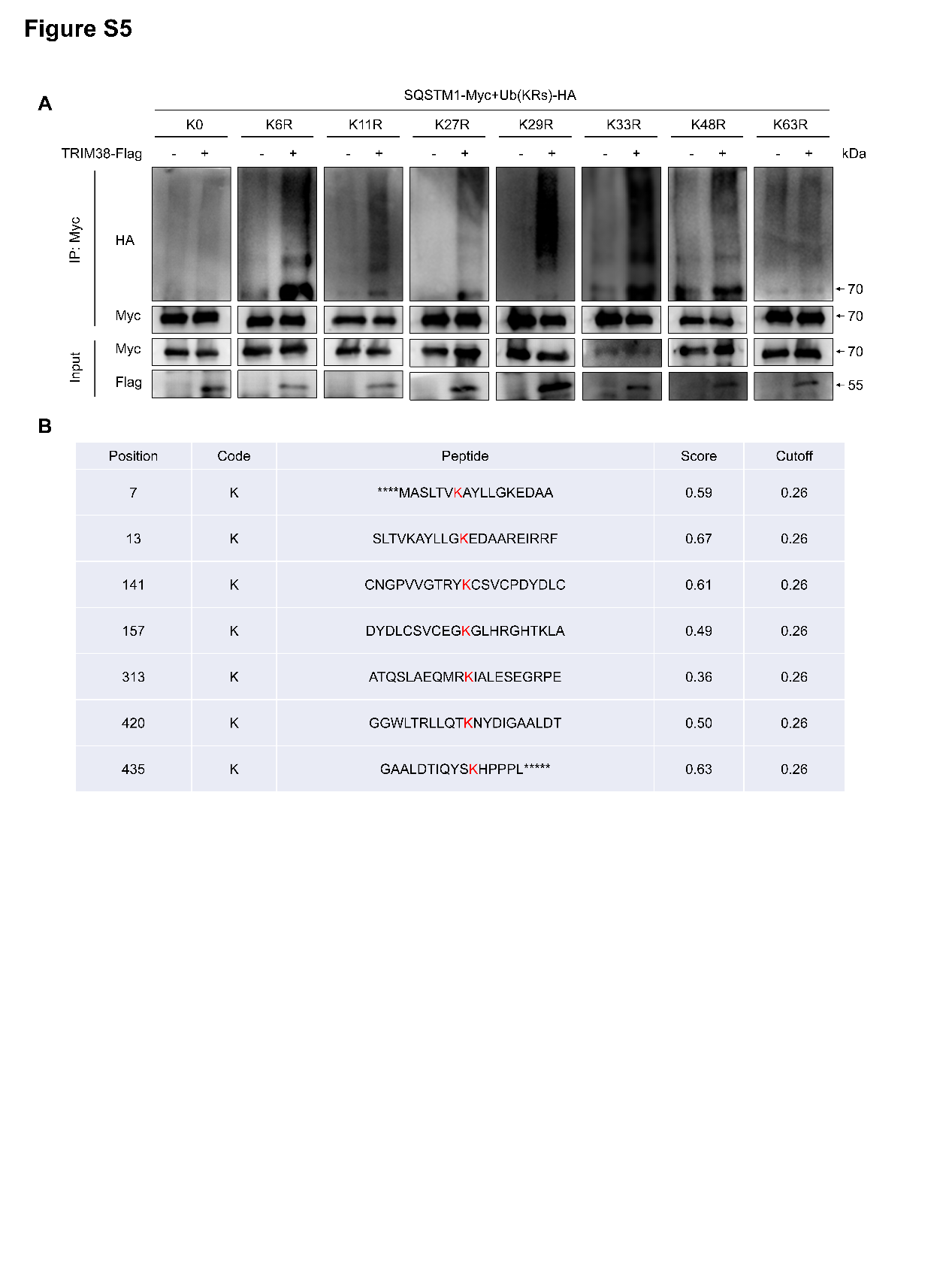
**

**Supplementary Figure 5. TRIM38 promotes SQSTM1 K63 polyubiquitination at K420 residue. A.** Immunoprecipitation analysis of lysates from HEK293T cells after transient co-transfection of Myc-SQSTM1, Flag-TRIM38, and series of HA-Ub mutant. **B.** Potential ubiquitinated residues on SQSTM1 predicted by GPS-Uber.


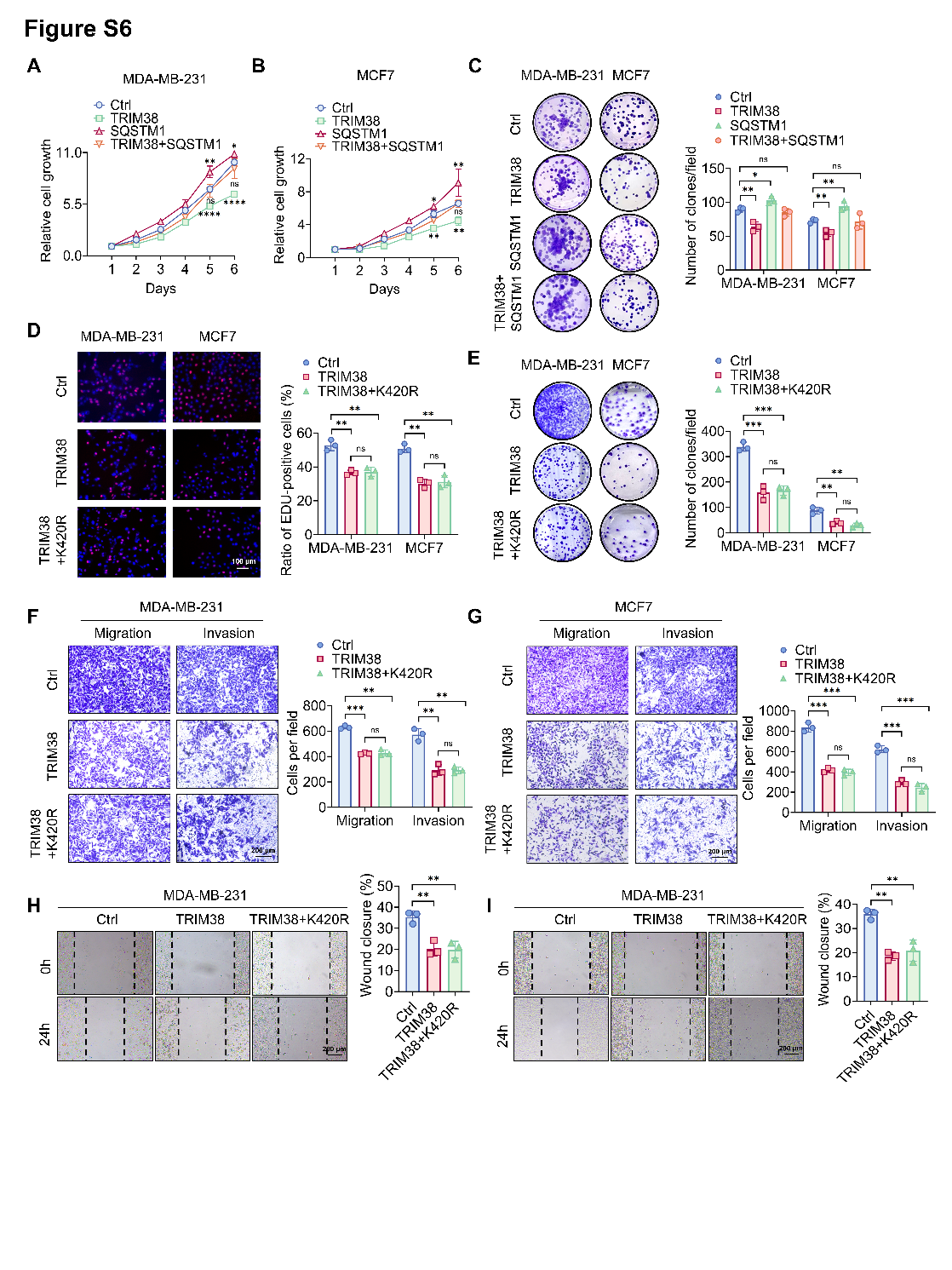


**Supplementary Figure 6. Tumor-suppressing effect of TRIM38 can be reversed by SQSTM1 but not SQSTM1 K420R. A-B.** MTT assays for cell proliferation of MDA-MB-231 **(A)** and MCF7 **(B)** across experimental groups. **C.** Cell plate colony formation assays to evaluate cell proliferation conducted across experimental groups in MDA-MB-231 and MCF7 cells. **D.** EdU assays for cell proliferation of MDA-MB-231 and MCF7 across experimental groups. **E.** Cell plate colony formation assays across experimental groups in MDA-MB-231 and MCF7 cells. **F-G.** Transwell assays for cell migration and invasion performed in MDA-MB-231 **(F)** and MCF7 **(G)** across experimental groups. **H-I.** Wound healing analysis of MDA-MB-231 (**H**) and MCF7 (**I**) cells transfected with specified experimental constructs. (mean ±SD, two-tailed t-test, *p < 0.05; **p < 0.01; ***p < 0.001; ****p<0.0001; n = 3). All experiments were repeated at least thrice and the results of representative experiments are shown.


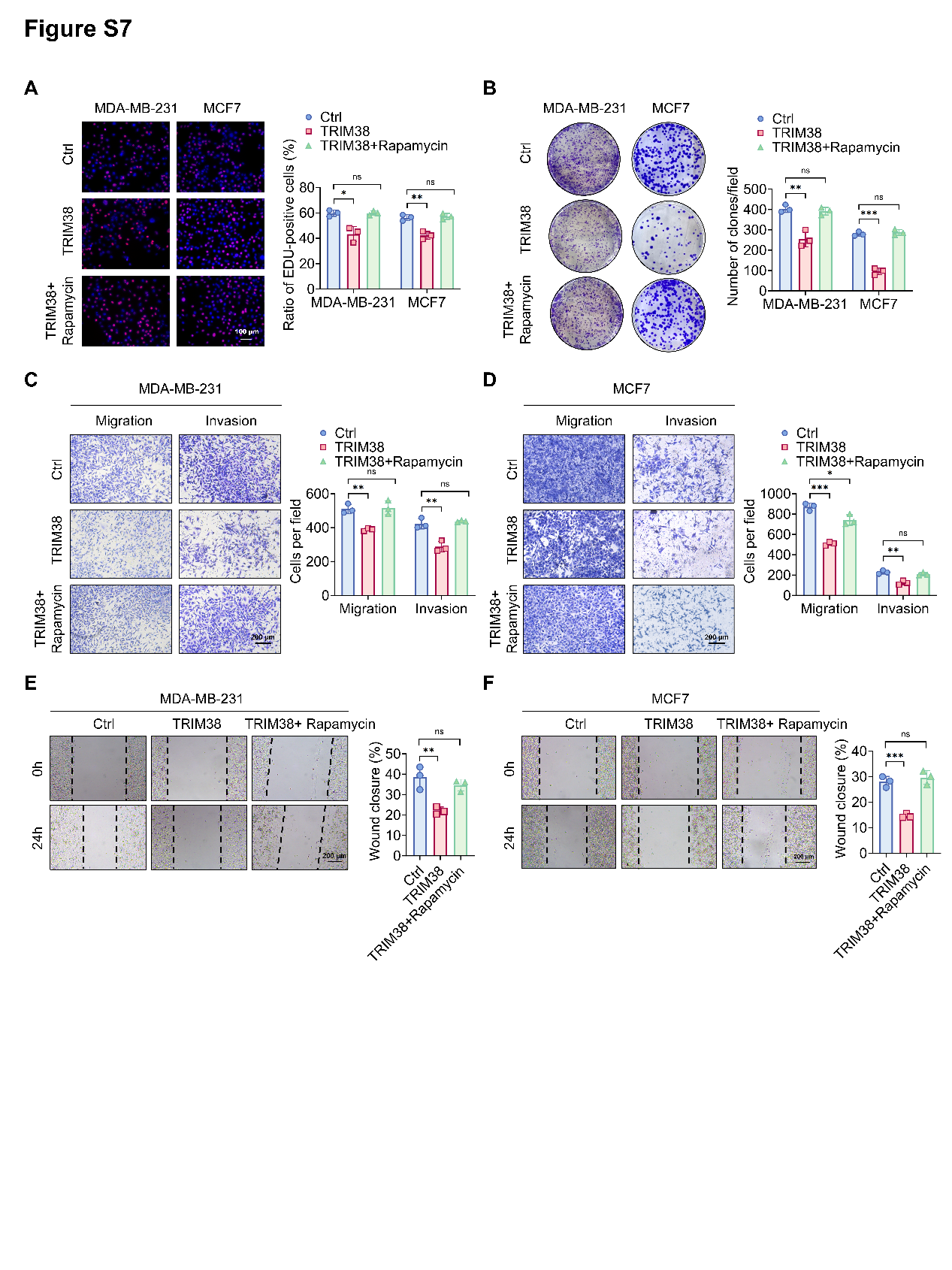


**Supplementary Figure 7 Tumor-suppressing effect of TRIM38 can be reversed by autophagy agonist rapamycin. A.** EdU assays for cell proliferation of MDA-MB-231 and MCF7 treated with rapamycin(200nM) for 24h after 24h-transfection of above plasmids. **B.** Cell plate colony formation assays to evaluate cell proliferation conducted across experimental groups in MDA-MB-231 and MCF7 cells. **C-D.** Transwell assays for cell migration and invasion performed in MDA-MB-231 **(C)** and MCF7 **(D)** across experimental groups. **E-F.** Wound healing assays for cell migration performed in MDA-MB-231 **(E)** and MCF7 **(F)** across experimental groups. (mean ±SD, two-tailed t-test, *p < 0.05; **p < 0.01; ***p < 0.001). All experiments were repeated at least thrice and the results of representative experiments are shown.


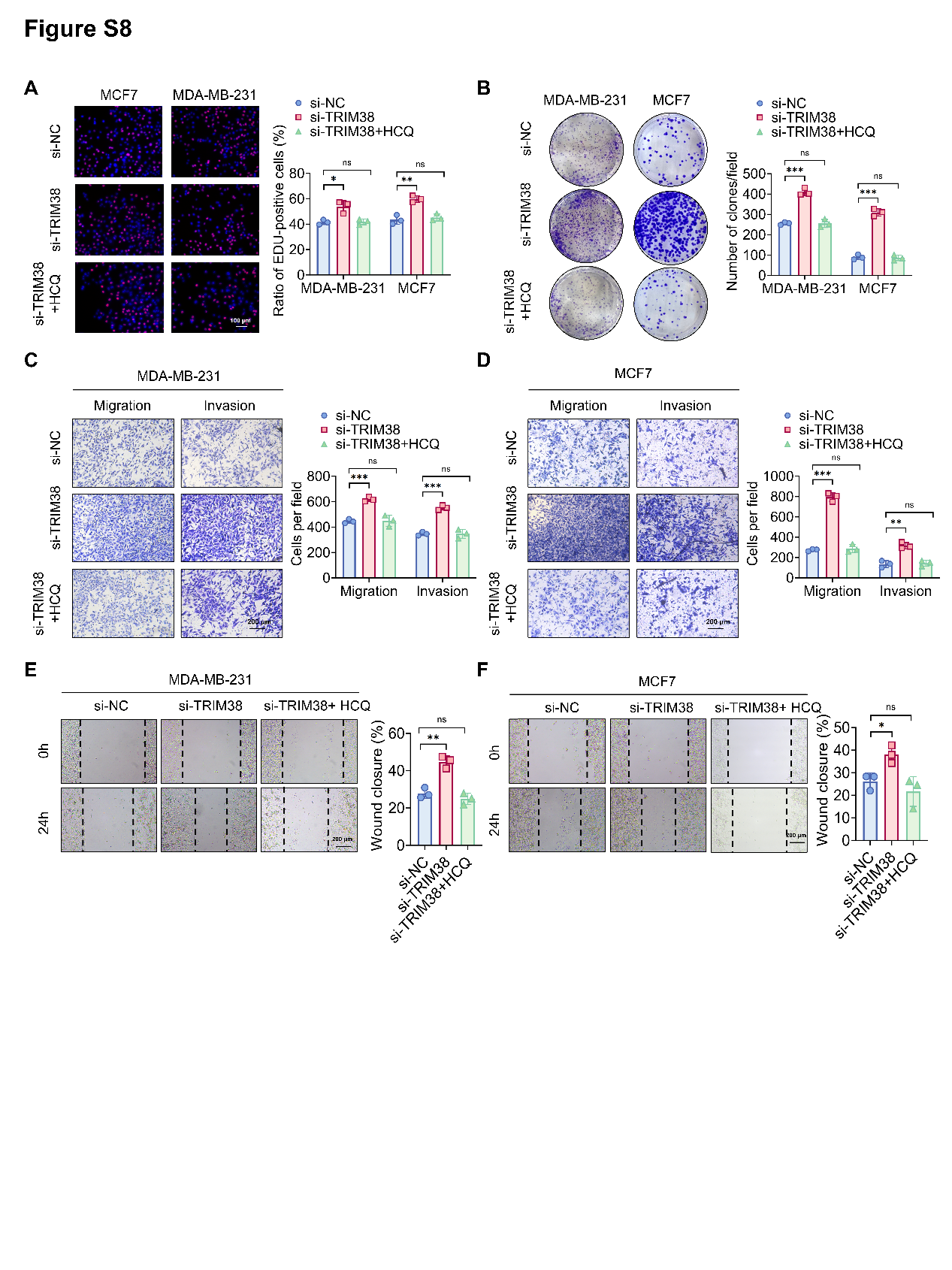


**Supplementary Figure 8. Autophagy blockade by HCQ reverses the oncogenic effects of TRIM38 deficiency. A.** EdU assays for cell proliferation of MDA-MB-231 and MCF7 treated with HCQ (20μM) for 24h after 24h-transfection of above si-RNAs. **B.** Cell plate colony formation assays to evaluate cell proliferation conducted across experimental groups in MDA-MB-231 and MCF7 cells. **C-D.** Transwell assays for cell migration and invasion performed in MDA-MB-231 **(C)** and MCF7 **(D)** across experimental groups. **E-F.** Wound healing assays for cell migration performed in MDA-MB-231 **(E)** and MCF7 **(F)** across experimental groups. (mean ±SD, two-tailed t-test, *p < 0.05; **p < 0.01; ***p < 0.001). All experiments were repeated at least thrice and the results of representative experiments are shown.

**Supplementary Tables**

**Supplementary Table 1 Key resources used in this study**

| **REAGENT or RESOURCE** | **SOURCE** | | **IDENTIFIER** |
| --- | --- | --- | --- |
| **Antibodies** |  | |  |
| anti-TRIM38  anti-TRIM38  anti-SQSTM1  anti-LC3  anti-Phospho-ULK1 (Ser758)  anti-Beclin1  anti-β-actin  anti-Ubiquitin  anti-Ubiquitin (K48-linked)  anti-Ubiquitin (K63-linked)  anti-Flag  anti-HA  anti-Myc  anti-His  anti-mouse IgG  anti-rabbit IgG  FITC Labeled Goat Anti-Rabbit IgG  Rhodamine Labeled Goat Anti-Mouse IgG | Abmart  Invitrogen  Proteintech  Proteintech  Proteintech  WanleiBio  Proteintech  Santa Cruz Biotechnology  Abcam  Abcam  Invitrogen  Sigma-Aldrich  Bethyl Laboratories  Beyotime  Affinity  Affinity  ZSGB-BIO  ZSGB-BIO | | MG825969M  MA5-26235  18420-1-AP  14600-1-AP  82776-8-RR  Wl02508  66009-1-Ig  sc-8017  ab140601  ab179434  PA1-984B  H6908  A190-105A  AF2870  #7076  #7074  ZF-0311  ZF-0313 |
| **Reagents** |  | |  |
| MG132  Chloroquine  Hydroxychloroquine  Rapamycin  Cycloheximide  Bafilomycin A1  3-MA  Protein A/G | Sigma-Aldrich  MCE  MCE  MCE  Selleck  Selleck  Selleck  Santa Cruz Biotechnology | | 474790  HY-17589A  HY-W031727  HY-10219  S7418  S1413  S2767  sc-2003 |
| **Critical Commercial Assays** |  | |  |
| BCA Protein Assay Kit | Merck Millipore | | 71285-3 |
| Cell lysis buffer for Western and IP | Beyotime Biotechnology | | P0013 |
| KOD-Plus-Mutagenesis kit | Toyobo | | SMK-101 |
| TRIzol | Invitrogen | | 15596018CN |
| Evo M-MLV RT Premix for qPCR  Hieff qPCR SYBR Green Master Mix (No Rox)  ECL detection system  EdU cell proliferation assay kit  Matrigel  IHC reagent kit  Diaminobenzidine  DAPI | ACCURATE BIOTECHNOLOGY  Yeasen Biotechnology (Shanghai)  Merck Millipore  RiboBio  Corning Incorporated  ZSGB-BIO  ZSGB-BIO  Servicebio | | AG11706  11201ES  WBKLS0500  C10310-1  356234  PV 9000  ZLI-9018  G1012 |
| **Experimental Models: Cell Lines** |  | |  |
| MDA-MB-231 | American Type Culture  Collection | | N/A |
| MCF7 | American Type Culture  Collection | | N/A |
| HEK293T cells | American Type Culture  Collection | | N/A |
| **Experimental Models: Organisms/Strains** |  | |  |
| Balb/c-nude mice | Beijing Vital River Laboratory Animal Technology | N/A | |

**Oligonucleotides**

| hTRIM38 | F | TTTGAGCAGGAGTTGGGC |
| --- | --- | --- |
|  | R | GCTTGGAAACATTGCA |
| hSQSTM1 | F | AGGCGCACTACCGCGAT |
|  | R | CGTCACTGGAAAAGGCAACC |
| hβ-actin | F | CATGTACGTTGCTATCCAGGC |
|  | R | CTCCTTAATGTCACGCACGAT |
| si-NC | S | UUCUCCGAACGUGUCACGUTT |
|  | AS | ACGUGACACGUUCGGAGAATT |
| si-1814 | S | GGCCCUAUUUCCAGGUUUATT |
|  | AS | UAAACCUGGAAAUAGGGCCTT |
| si-915 | S | GUCCACAGCAAUGCGAAUATT |
|  | AS | UAUUCGCAUUGCUGUGGACTT |

**Generating mutant plasmid**

| TRIM38 C16A | F | GTCTCCATCTGCCTGAGC |
| --- | --- | --- |
|  | R | GGTGGCTTCCTCCATCATC |
| TRIM38 N | F | TTCAACAAGAGCTGTGGTGTG |
|  | R | AAAGATATCCTGGATTACAAGGATGACGAC |
| TRIM38 C | F | GACGTATGCCAGGGCTACAAG |
|  | R | CATGAGCGGCCGCG |
| SQSTM1 △PB1 | F | CATGGTGGCGAATTCCACCA |
|  | R | GCCATGTCCTACGTGAAGGATGA |
| SQSTM1 △ZZ | F | CATGTTGCGGGGCGCCTC |
|  | R | CCCAGCCCCTTCGGG |
| SQSTM1 △TB | F | ACCAGAAGCTGATTCTGCCG |
|  | R | GAAGTTGATATCGATGTGGAGCACG |
| SQSTM1 △LIR | F | CTCGGACTCCAAGGCGATC |
|  | R | CTGTCTTCAAAAGAAGTGGACCCG |
| SQSTM1 △UBA | F | TGGGTACAAGGCAGCTTCCT |
|  | R | CTCGAGGAACAAAAACTAATATCAGAGGAAG |

**Supplementary Table 2 Baseline patient demographics and clinicopathologic characteristics of patients in 157 breast cancer patients.**

| **Characteristics** | **Number of patients**  **(n=157)** |
| --- | --- |
| **Year of diagnosis** |  |
| 2007 | 1 |
| 2008 | 7 |
| 2009 | 12 |
| 2011 | 6 |
| 2012 | 124 |
| 2013 | 6 |
| 2014 | 1 |
| **Age** |  |
| ≦50 | 90 |
| >50 | 67 |
| **Tumor size** |  |
| <=2cm | 95 |
| >2cm | 62 |
| **LN metastasis** |  |
| 0 | 104 |
| 1-3 | 31 |
| >3 | 22 |
| unknow | 1 |
| **Histologic Grade** |  |
| G1 | 12 |
| G2 | 88 |
| G3 | 38 |
| Unknown | 19 |
| **Stage** |  |
| I A | 55 |
| II A | 40 |
| II B | 12 |
| III A | 15 |
| III B | 6 |
| III C | 8 |
| Unknown | 21 |
| **ER** |  |
| Negative | 45 |
| Positive | 112 |
| **PR** |  |
| Negative | 58 |
| Positive | 99 |
| **HER-2** |  |
| Negative | 147 |
| Positive | 10 |
| **Ki67** |  |
| <=14% | 51 |
| >14% | 106 |
| **Surgical approaches** |  |
| Left breast cancer modified radical mastectomy | 72 |
| Right breast cancer modified radical mastectomy | 58 |
| Bilateral breast cancer modified radical mastectomy | 3 |
| Breast-conserving surgery | 2 |
| Unknown | 22 |
| **Undergo chemotherapy** |  |
| Yes | 103 |
| No | 32 |
| Unknown | 22 |

**Supplementary Table 3 Correlation between TRIM38 expression and clinical parameters** **in 157 breast cancer patients**

| Characteristics | TRIM38-low (n=73) ^#^ | | TRIM38-high (n=84) ^#^ | | *P* value ^*^ |
| --- | --- | --- | --- | --- | --- |
| **Age** | |  |  |  | |
| ≦50 | | 49(67.1%) | 41(48.8%) | **0.021** | |
| >50 | | 24(32.9%) | 43(51.2%) |  |  |
| **Tumor size** | |  |  |  | |
| <=2cm | | 36(49.3%) | 59(70.2%) | **0.007** | |
| >2cm | | 37(50.7%) | 25(29.8%) |  |  |
| **LN metastasis** | |  |  |  | |
| 0 | | 49(67.1%) | 54(65.1%) | 0.248 | |
| 1-3 | | 17(23.3%) | 14(16.8%) |  |  |
| >3 | | 7(9.6%) | 15(17.8%) |  |  |
| Unknown | | 0 | 1 |  |  |
| **Histologic Grade** | |  |  |  | |
| G1 | | 5(8.3%) | 7(9.0%) | 0.851 | |
| G2 | | 37(61.7%) | 51(65.4%) |  |  |
| G3 | | 18(30.0%) | 20(25.6%) |  |  |
| Unknown | | 13 | 6 |  |  |
| **ER** | |  |  |  | |
| Negative | | 28(38.4%) | 17(20.2%) | **0.012** | |
| Positive | | 45(61.6%) | 67(79.8%) |  |  |
| **PR** | |  |  |  | |
| Negative | | 33(45.2%) | 25(29.8%) | **0.046** | |
| Positive | | 40(54.8%) | 59(70.2%) |  |  |
| **HER-2** | |  |  |  | |
| Negative | | 70(95.5%) | 77(91.7%) |  | |
| Positive | | 3(4.1%) | 7(8.3%) | 0.280 | |
| **Ki67** | |  |  |  | |
| <=14% | | 17(23.3%) | 34(40.5%) |  | |
| >14% | | 56(76.7%) | 50(59.5%) | **0.022** | |

**. P* values are listed for a χ2 test for categorical variables.

#. The expression level of TRIM38 was assessed using IHC scores within breast carcinoma regions.

**Supplementary Table 4 Overall survival of patients involved in this study**

| Variable | Univariate analysis |  | Multivariate analysis | |  |
| --- | --- | --- | --- | --- | --- |
|  | HR (95% CI) | *P** | HR (95% CI) | *P* | |
| Age (>=50 vs. <50) | 2.24(0.97-5.18) | **0.059** | 2.79(1.08-7.22) | **0.035** | |
| Histologic Grade |  |  |  |  | |
| G2 vs. G1 | 2.00(0.25-15.72) | 0.513 | 1.71(0.20-14.93) | 0.627 | |
| G3 vs. G1 | 4.67(0.57-36.02) | **0.149** | 3.84(0.40-36.99) | 0.244 | |
| Tumor Size (>2 cm vs. <=2cm) | 1.43(0.62-3.25) | 0.394 | - | - | |
| LN metastasis |  |  |  |  | |
| 1-3 vs. 0 | 1.15(0.37-3.52) | 0.812 | 1.53(0.46-5.64) | 0.486 | |
| >3 vs. 0 | 2.52(0.95-6.70) | **0.063** | 6.07(1.94-19.02) | **0.002** | |
| ER Status (pos vs. neg) | 0.42(0.18-0.99) | **0.047** | 0.79(0.08-7.04) | 0.830 | |
| PR Status (pos vs. neg) | 0.49(0.21-1.16) | **0.105** | 1.07(0.13-8.87) | 0.951 | |
| Her-2 Status (pos vs. neg) | 1.78(0.41-7.62) | 0.447 | - | - | |
| Ki67 Status (<=14% vs. >14%) | 1.12(0.46-2.72) | 0.802 | - |  | |
| TRIM38 expression | 0.41(0.17-0.99) | **0.048** | 0.21(0.07-0.61) | **0.004** | |

**Supplementary Table 5 Distant disease-free survival of patients involved in this study**

| Variable | Univariate analysis |  | Multivariate analysis | |  |
| --- | --- | --- | --- | --- | --- |
|  | HR (95% CI) | P* | HR (95% CI) | P | |
| Age (>=50 vs. <50) | 1.63(0.82-3.23) | 0.166 | - | **-** | |
| Histologic Grade |  |  |  |  | |
| G2 vs. G1 | 4.23(0.56-32.11) | 0.162 | 4.90(0.64-37.86) | 0.127 | |
| G3 vs. G1 | 6.77(0.86-52.92) | **0.068** | 7.92(0.98-63.77) | **0.052** | |
| Tumor Size (>2cm vs. <=2cm) | 1.88(0.95-3.75) | **0.072** | 1.20(0.56-2.53) | 0.646 | |
| LN metastasis |  |  |  |  | |
| 1-3 vs. 0 | 1.72(0.75-3.96) | 0.203 | 2.08(0.85-5.07) | 0.107 | |
| >3 vs. 0 | 1.98(0.82-4.81) | **0.130** | 3.82(1.43-10.21) | **0.007** | |
| ER Status (pos vs. neg) | 0.77(0.38-1.59) | 0.492 | - | - | |
| PR Status (pos vs. neg) | 0.95(0.47-1.92) | 0.880 | - | - | |
| Her-2 Status (pos vs. neg) | 1.42(0.33-6.06) | 0.637 | - | - | |
| Ki67 Status (<=14% vs. >14%) | 0.77(0.38-1.55) | 0.464 |  |  | |
| TRIM38 expression | 0.41(0.19-0.86) | **0.018** | 0.25(0.10-0.59) | **0.002** | |

*The above independent variables are closely related to the survival of patients clinically, which are considered to be included in the model. Therefore, the threshold of P value is appropriately relaxed to 0.15 to avoid missing important factors.
